# Description of six new large species of *Argentinomyia* Lynch-Arribálzaga, 1891 and redescription of *Talahua fervida* (Fluke, 1945) (Diptera, Syrphidae, Syrphinae)

**DOI:** 10.3897/zookeys.929.37666

**Published:** 2020-04-22

**Authors:** Augusto L. Montoya, Marta Wolff

**Affiliations:** 1 Grupo de Entomología, Universidad de Antioquia, Calle 67 # 53-108, Medellín, Colombia Universidad de Antioquia Medellín Colombia

**Keywords:** Endemism, flower flies, hover flies, Neotropical diversity, Mesoamerica, Tropical Andes

## Abstract

The morphological similarities between five new large *Argentinomyia* species and *Talahua fervida* Fluke are characterized and presented. Six new species of *Argentinomyia* (10–12 mm long) are described: *Argentinomyia andina* Montoya & Wolff, **sp. nov.** (Colombia), *Argentinomyia choachi* Montoya, **sp. nov.** (Colombia), *Argentinomyia quimbaya* Montoya & Wolff, **sp. nov.** (Colombia), *Argentinomyia huitepecensis* Montoya, **sp. nov.** (México), *Argentinomyia puntarena* Montoya, **sp. nov.** (Costa Rica), and *Argentinomyia talamanca* Thompson, **sp. nov.** (Costa Rica). The genus *Talahua* Fluke is re-diagnosed and, *Talahua fervida* redescribed. A taxonomic key and a comparison of diagnostic characters are presented. Photographs of head, abdominal and wing maculae patterns, as well as illustrations of male genitalia are provided for species identification.

## Introduction

Flower flies or hoverflies (Syrphidae) are one of the most diverse families of Diptera with more than 6100 described species worldwide, and ca. 1560 species distributed in the Neotropical region (Thompson et al. 2010; Pape and Evenhuis 2018). Currently, the family is subdivided into four subfamilies: Syrphinae, Pipizinae, Eristalinae, and Microdontinae (Mengual 2015). Syrphinae comprises more than 30% of worldwide diversity of the family and contains mostly species with larvae that feed on soft-body arthropods (Rojo et al. 2003). Within Syrphinae, the tribe Bacchini comprises 332 species worldwide (13 genera) and more than 83 Neotropical species classified into seven genera: *Melanostoma* Schiner, 1860 is present in all biogeographic regions (Haarto and Ståhls 2014); in the Neotropics, only *Melanostoma bellum* Giglio-Tos, 1892 is found in Chiapas, México, but may also occur in the adjacent highlands of Guatemala (Thompson et al. 2010). *Platycheirus* Lepeletier & Serville, 1828 and *Xanthandrus* Verrall, 1901 are widely distributed in the Neotropics and other biogeographic regions (Vockeroth 1990; Borges and Pamplona 2003; Mengual et al. 2008). Genera *Argentinomyia* Lynch-Arribálzaga, 1891, *Leucopodella* Hull, 1949, *Tuberculanostoma* Fluke, 1943, and *Talahua* Fluke, 1945 are Neotropical endemics and reach the highest diversity in the highlands of the Tropical Andes (Fluke 1943, 1945, 1957; Thompson 1981, 1999), with some species of *Argentinomyia* and *Leucopodella* extending to Central America.

*Argentinomyia* contains 27 valid species distributed from the cloud forests in Northern Central America to low and middle elevations in the Caribbean and Galápagos Islands. The genus is also found in cold Andean forests and Páramo ecosystems in the Tropical Andes, and in lowlands in southeastern of South America. Even though extensive sampling has been done, the genus is apparently absent in the Chilean subregion and, has not been registered in Surinam (Thompson 1999; Thompson et al. 2010; Reemer 2010). Adults of *Argentinomyia* are common flower visitors in pristine ecosystems, whilst immature stages are unknown.

*Argentinomyia* is distinguished from other genera of Bacchini by the combination of: 1) long antenna, with scape much longer than broad; 2) basoflagellomere oval or slightly elongate; 3) face straight in profile, not produced anteriorly, generally with pollinosity broadly punctuate, tubercle low, usually with transverse grooves dorsally or pollinosity broadly punctuate; 4) metacoxa without posteromedial pile on apical angle; 5) abdomen dark colored, often with variously shaped light-colored yellow, orange to silvery-grey pollinose paired maculae; triangular to quadrate or oval markings on 2^nd^ to 4^th^ abdominal tergite, sometimes including a small macula on 5^th^ tergite, and 6) male genitalia normal size, superior lobes triangular to rectangular, irregular in shape and cercus short (Fluke 1945, 1957; Thompson 1999; Huo 2014; Thompson and Skevington 2014).

*Talahua* is a small Neotropical genus that inhabits the highlands of Colombia and Ecuador (Fluke 1945; Montoya et al. 2012; Montoya 2016; Marín-Armijos et al. 2017). The genus was originally established by Fluke (1945) as subgenus of *Melanostoma*, including the only species Melanostoma (Talahua) fervida Fluke, 1945. In a subsequent study, Fluke (1957) gave *Talahua* full generic status based on the study of male genitalia. Later, Thompson et al. (1976) also considered *Talahua* a valid genus. In 1999, Thompson proposed to transfer the aberrant species, *Melanostoma palliatum* Fluke, 1945 to *Talahua*, despite the species being previously considered part of *Xanthandrus* (Thompson et al. 1976). In the revision of the Neotropical *Xanthandrus*, Borges and Pamplona (2003) considered *M. palliatum* as part of *Xanthandrus*, and consequently, *Talahua* was again recognized as monotypic (Borges and Pamplona 2003; Marín-Armijos et al. 2017; Thompson and Skevington 2014).

*Talahua* can be distinguished from other genera of Bacchini largely by the following combination of characters: 1) antennae relatively short, scape broader than long, nearly equal to pedicel; 2) basoflagellomere large, slightly oval and apically rounded; 3) face slightly receding to perpendicular with a well-rounded tubercle, never with transverse grooves dorsally along tubercle or broadly punctuate; 4) metacoxa with a tuft of pile at posteromedial apical angle; 5) abdomen elongated or with parallel sides, with four to five pairs of large rounded to triangular markings on the terga, always with small macula on 5^th^ tergum; and 6) male genitalia greatly enlarged, with superior lobes, and cerci elongated, surstyli three to four times longer than broad (Fluke 1945, 1957; Thompson 1999; Thompson and Skevington 2014).

Extensive sampling in the cloud forest, high-Andean and Páramo ecosystems in Mesoamerica (México and Costa Rica) and Tropical Andes (Colombia and Ecuador) in the last twenty-five years resulted in the discovery of several new *Argentinomyia* species including six species, large in body size, that are similar in appearance to *Talahua fervida*. Therefore, we take the opportunity to describe the new species and provide a full redescription for *T. fervida*, as well as a taxonomic key, photographs, illustrations, and a comparison of morphological diagnostic characters, including distributional maps to all species.

## Material and methods

Syrphidae -specific characters used in the key, descriptions, and drawings largely follows the terminology established by Thompson (1999), Thompson et al. (2010), and Cumming and Wood (2017). Figures of some characters employed in the key correspond to those treated in the chapter of Syrphidae in the Manual of Central America Diptera (Thompson et al. 2010) and are indicated by the abbreviation "MCAD". The specimens were determined to genus level using the keys of Thompson et al. (2010), Huo (2014) and Thompson and Skevington (2014). Our new species were compared with type specimens of twenty-seven *Argentinomyia* species and one of *Talahua* deposited in the AMNH, BMNH, USNM, and WIRC collections (Suppl. material 1), including the study of the original descriptions (Fluke 1945). A complete revision of the genus is in preparation and will be published soon. Recognition of the new species was facilitated by examination and comparison of the reference material of *Argentinomyia* identified by F. C. Thompson in the USNM (Smithsonian Institution). The acronyms used for the collections examined are as follows (curators’ names in parentheses):

**AMNH** American Museum of Natural History, New York, USA (David Grimaldi)

**CEUA** Colección de Entomología Universidad de Antioquia, Medellín, Colombia (Marta Wolff)

**ECO-TAP-E** Colección Entomológica de la Unidad San Cristóbal de las Casas de El Colegio de la Frontera Sur, México (Philippe Sagot and Rémy Vandame)

**IAvH** Instituto de Investigación de Recursos Biológicos Alexander von Humboldt, Villa de Leyva, Colombia (John César Neita-Moreno)

**INBio** Instituto Nacional de Biodiversidad, Heredia, Costa Rica (Manuel Zumbado)

**QCAZ** Departamento de Biología, Pontifica Universidad Católica del Ecuador, Quito, Ecuador (Álvaro Barragán)

**UNAB** Museo Entomológico de la Facultad de Agronomía, Universidad Nacional de Colombia, Bogotá, Colombia (Francisco Serna and Erika Vergara)

**USNM** National Museum of Natural History, Washington, D.C., USA (Torsten Dikow)

**WIRC** Wisconsin Insect Research Collection, Department of Entomology, University of Wisconsin, Madison, USA (Steven Krauth)

The type series of the new species is comprised of dry pinned material deposited in the CEUA, USNM, INBio, and ECO-TAP-E.

To illustrate the morphological variation of the herein described species, habitus photographs were created from a series of images taken at different focal depths with a digital camera Olympus OM-D (Olympus Raw Image file in .ORF) using the facilities of the Diptera Collection, Department of Entomology (https://naturalhistory.si.edu/research/entomology/collections-overview/diptera-collection) at the USNM. Additional photos were taken using a Moticam 3.0 megapixel DFC500 digital camera attached to an Olympus SZX7 stereomicroscope. Final images were combined using the HeliconFocus Pro (version 6.7.1) stacking software. The scale bar was added in Photoshop according to the camera focal aperture used when the photo was taken. Editing was conducted in Adobe Photoshop CC, and the final image plates were prepared in Illustrator CC.

Body length was measured from frons to the posterior end of the abdomen; wing length was measured from wing insertion to the apex of the wing. Measurements were made using a Zeiss Stemi 2000-C stereomicroscope (magnification 6.5–115×) equipped with a stereoscope grid. Measurements of antennal segments are approximations based on the mid-line of the inner surface and are presented in the ratio format scape:pedicel:flagellomere.

For the study of the male genitalia, the structure was dissected. The genitalia were cleared in a KOH solution (approximately 10%) boiling at 37 °C for 10 to 15 minutes. Schema of internal structures were illustrated from digital images taken through the stereomicroscopes. Additional, sketches were produced with a camera Lucida attached to the stereomicroscope. Final drawings were prepared by tracing and vectorizing in Adobe Illustrator CC, and pile was omitted.

The new species are described from males and females collected together in at least one locality, and sexual dimorphic variation reported. Only *Argentinomyia choachi* Montoya sp. nov. is described from a single female because it markedly differs in the morphological characters from the other species.

With the aim of spanning the entire known distribution of included species, original label information was compiled in a Darwin Core standard-compliant data. Distributional maps were generated using the software QGIS desktop 2.2.0 and an excel .csv file (comma delimited) to plot presence. A digital file with an elevation model (SRTM30 CGIAR-SRTM with 30 seconds resolution) was used in addition to a shapefile with the biogeographic provinces proposed by Morrone (2014) and digitalized by Löwenberg-Neto (2015) for the Neotropics.

## Description of new species

The new *Argentinomyia* species described here are characterized by the scutellum with a deep groove next to the rim (emarginate), face with a well-rounded tubercle, never with transversal grooves dorsally along tubercle or broadly punctuate, metacoxa with a tuft of pile at posteromedial apical angle, wing generally with a brownish macula extensively covering the apex of cells r and m or hyaline, and abdomen with large markings on the terga. The new species are superficially similar to *Talahua fervida*, differing in the male genitalia.

### Identification key to the large (10–12 mm long) species of *Argentinomyia*

The new key was modified based on characters provided by Thompson (1999), Thompson et al. (2010), Huo (2014), Thompson and Skevington (2014) and Ramage et al. (2018).

**Table:** 

1	Postpronotum pilose (MCAD fig. 30); male abdomen with four unmodified pregenital segments; tergum 5 usually not visible in dorsal view (MCAD figs 1, 4) (subfamilies Eristalinae, Microdontinae and Pipizinae)	**other flower flies**
–	Postpronotum bare; male abdomen with five unmodified pregenital segments; tergum 5 visible in dorsal view (MCAD figs 53-61) (subfamily Syrphinae)	**2**
2	Face and/or scutellum partially yellow or yellowish-brown in background color, aedeagus two-segmented	**other Syrphinae genera**
–	Face and scutellum entirely black in background color (some species with partly pale face or scutellum), aedeagus unsegmented (Bacchini)	**3**
3	Abdomen petiolate, distinctly narrower than thorax (MCAD figs 59, 60); face usually without tubercle, flat, straight or convex	***Leucopodella* Hull**
–	Abdomen parallel-sided or narrowly oval (MCAD figs 55, 58); face with tubercle	**4**
4	Antennal cavity broadly confluent (MCAD fig. 24); metathoracic pleuron with several fine subappressed pile ventral to spiracle; katepisternum with pile patches broadly separated posteriorly, joined anteriorly	***Xanthandrus* Verrall**
–	Antennal cavity broadly separated (MCAD fig. 23); metathoracic pleuron bare; katepisternum with pile patches usually broadly separated throughout	**5**
5	Metasternum greatly reduced to a small diamond (MCAD fig. 34; Haarto and Ståhls 2014: 95, fig. 1A); face not produced below, with small tubercle, facial pruinosity neither punctate nor rippled (MCAD fig. 28)	***Melanostoma* Schiner**
–	Metasternum entire, not reduced (MCAD fig. 35; Haarto and Ståhls 2014: 95, fig. 1B); face variable, almost straight in profile with weak tubercle or moderately to strongly produced forward ventrally, sometimes with pruinescence forming punctuate or rippled pattern	**6**
6	Antennae shorter, scape broader than long, scape nearly equal to pedicel, basoflagellomere large, slightly oval and apically rounded (Fig. 12 A, B); face perpendicular with a well-rounded tubercle (Figs 12A, 13A, D); mesocoxa pilose posteriorly; male genitalia enlarged (Fluke 1957: 278, fig. 123)	***Talahua* Fluke**
–	Antenna elongate, scape longer than broad; basoflagellomere oval or elongate (MCAD fig. 22); face straight in profile, tubercle low (MCAD fig. 22); mesocoxa bare posteriorly; male genitalia normal size (*Argentinomyia* Lynch-Arribálzaga *sensu lato*)	**7**
7	Basoflagellomere oval or slightly elongate (MCAD fig. 22); face usually with transversal grooves dorsally along tubercle (MCAD fig. 23) and shine (bare) punctuate maculae laterally; scutellum without a deep groove next to the rim; metacoxa without a pile tuft at posteromedial apical angle; abdomen slightly spatulate, oval or with parallel sides, with triangular to quadrate or oval markings	**other species of *Argentinomyia*** (not treated here)
–	Basoflagellomere large, slightly oval and apically rounded (Fig. 1C, F); face with a well-rounded tubercle, never with transversal grooves dorsally along tubercle or broadly punctuate (Fig. 1A, D); scutellum with a deep groove next to the rim (emarginate) (Figs 1B, 1E, 12D); metacoxa with pile posteromedial on apical angle (Fig. 12C); abdomen elongated, with large markings on the terga, sometimes with a transverse fascia on the third or with a pair of small maculae in the basal corners of fifth tergum (Fig. 1B, E)	**8**
8	Antenna brown (Fig. 10A, C, D, F); alula and costal cell extensively microtrichose (Fig. 10B, C, D); femur extensively brown (Fig. 10C, F); second tergum with a pair of small maculae on basal 1/5 (Fig. 10B–E); male genitalia as Fig. 11A–C [Costa Rica]	***Argentinomyia talamanca* Thompson, sp. nov.**
–	Antenna orange ventrally; alula and costal cell bare; femur yellow on apical 1/3 or more; second tergum with broad macula	**9**
9	Legs extensively yellow (Fig. 8A–E); male genitalia as Fig. 9A–C [Colombia]	***Argentinomyia quimbaya* Montoya & Wolff, sp. nov.**
–	Legs black on basal 1/3 or more (Figs 1, 3, 4, and 6)	**10**
10	Abdomen with a transverse fascia on the third tergum (Fig. 4B, E); metacoxa black pilose (Fig. 4C, F); male genitalia as Fig. 5A–C [México]	***Argentinomyia huitepecensis* Montoya, sp. nov.**
–	Abdomen with a pair of large maculae on the third tergum, slightly touching each other toward the middle; metacoxa yellowish pilose (Figs 1, 3, 6)	**11**
11	Metafemur brown basally; coxa black; third and fourth tergum with a pair of quadrangular maculae (Fig. 3A–D) (male unknown) [Colombia]	***Argentinomyia choachi* Montoya, sp. nov.**
–	Metafemur yellow basally; coxa orange-brown; third and fourth tergum with a pair of triangular maculae (Figs 1, 6)	**12**
12	Face white pollinose and pilose (Fig. 6A, D); metafemur orange on basal 1/5 and apical 3/5, metatibia extensively brown, only orange brownish on basal 2/3 (Fig. 6C, F); fifth tergum without maculae (Fig. 6B, E); male genitalia as Fig. 7A, C [Costa Rica]	***Argentinomyia puntarena* Montoya, sp. nov.**
–	Face yellow pollinose and pilose (Fig. 1A, D); metafemur brown, only slightly orange on apical 1/6, tibiae yellow with a dark ring near the middle, more prominent on the metalegs (Fig. 1C, F); fifth tergum with a pair of small maculae in the basal corners (Fig. 1B, E); male genitalia as Fig. 2A–C [Colombia]	***Argentinomyia andina* Montoya & Wolff, sp. nov.**

#### Argentinomyia andina

Taxon classificationAnimaliaDipteraSyrphidae

Montoya & Wolff sp. nov.

1B1BC6E4-AE2C-5988-946C-2ABEE31DB109

http://zoobank.org/72FC4AEB-1E3D-4529-8DE2-E541D25207EB

Figures 1 , 2 , 15

##### Differential diagnosis.

Face yellow pollinose and pilose. Metafemur extensively brown, only slightly orange on apical 1/6. Tibiae yellow with a dark ring near the middle, more prominent on the metalegs. Third and fourth tergum with a pair of broad subquadrate maculae, reaching the lateral margin in their full width, fifth tergum with a pair of small maculae in the basal corners. *Argentinomyia puntarena* sp. nov. is similar to *A. andina* sp. nov., but differs in having the face white pollinose and pilose; metafemur orange on basal 1/5 and apical 3/5, metatibia extensively brown, only orange brownish on basal 2/3; fifth tergum without maculae.

##### Type locality.

Colombia, department of Antioquia, Sonsón municipality, Vereda Norí municipal rural settlement, Norí Mountain hill, forest, 05°48.580'N, 75°16.142'E, alt. 2896 m a.s.l.

##### Description.

**Figure 1.**
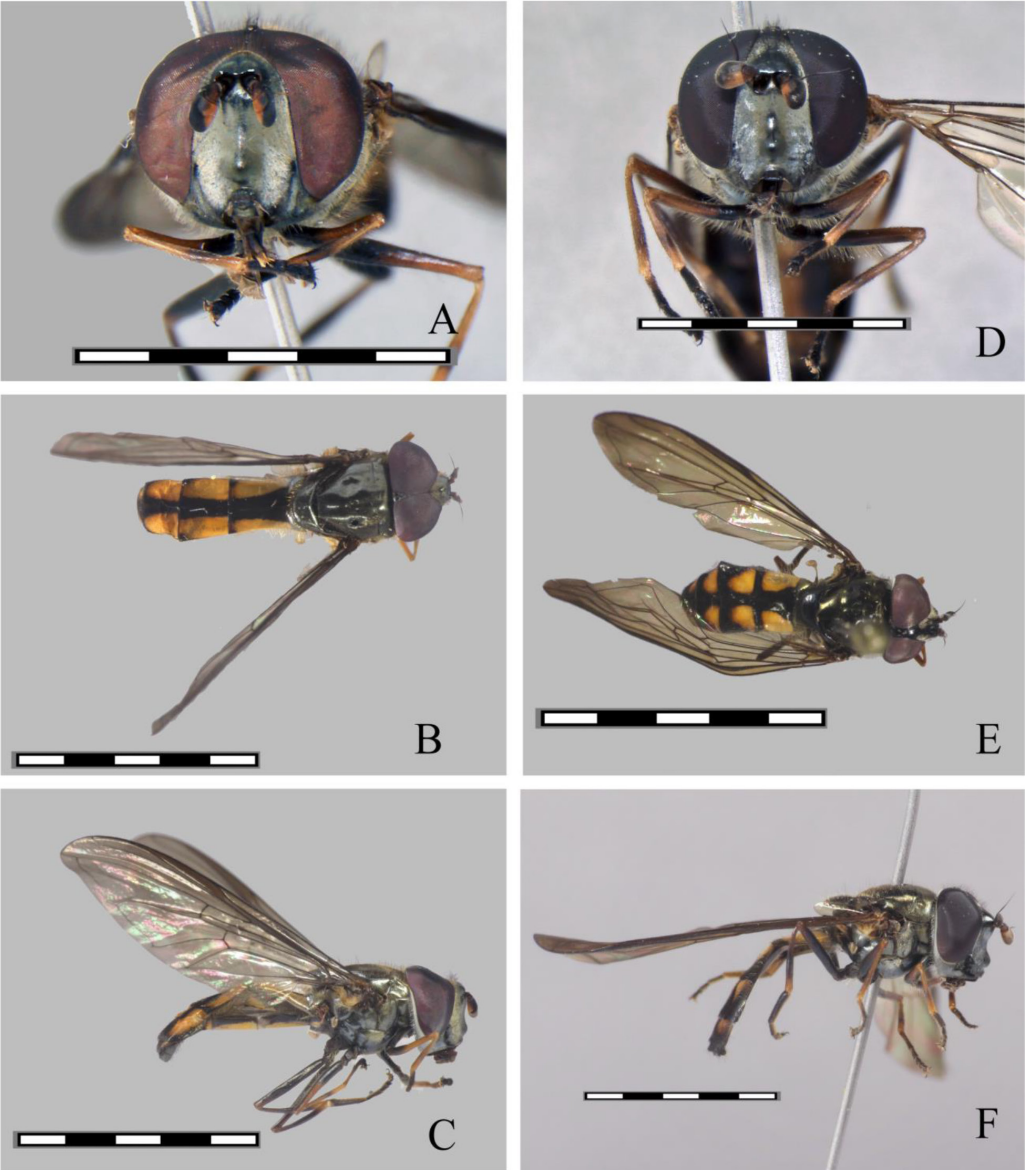
*Argentinomyia andina* sp. nov., male (CEUA 103551): **A** head, frontal, male **B** dorsal view **C** lateral view. Female (CEAU 69016): **D** head, frontal view **E** dorsal view **F** lateral view. Scale bars: 5 mm.

**Figure 2.**
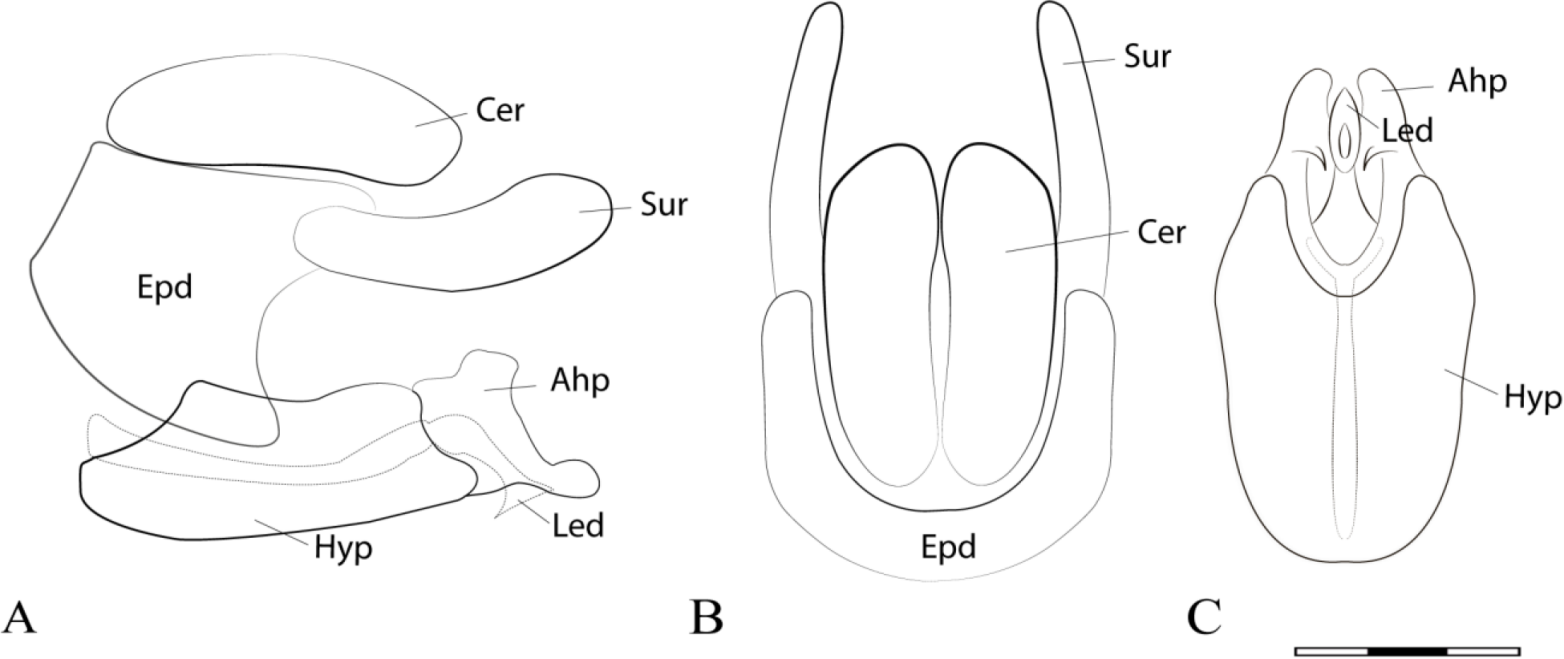
*Argentinomyia andina* sp. nov., male genitalia: **A** whole genitalia including epandrium, cercus, and surstylus, lateral view **B** epandrium, dorsal view **C** hypandrium, ventral view. Abbreviations used in male genitalia structures are as follows: **Ahp** = apex of hypandrium (superior lobes; **Cer** = cercus; **Epd** = epandrium; **Hyp** = hypandrium; **Led** = aedeagal lobe; **Sur** = surstyle. Scale bar: 0.05 mm.

**Male. Head** (Fig. 1A, C): Black metallic, covered with yellow pollinosity, oral tips, ocellar triangle, and a large triangular macula on the frons, yellow pilose, pile on front black, on gena and face golden yellow, on the occiput yellow except the dorsal pile black, frontal triangle coppery metallic. Antennae brown, orange-red ventrally, rounded, as long as wide, the lower basal corner of basoflagellomere, ratio 1.0:1.2:2.3, arista orange, dark brown toward the tip. **Thorax** (Fig. 1C). Black, the scutum shining, with iridescent to coppery yellow reflections, with two median brownish pollinose vittae on anterior half, pile mostly yellow, with long black pile before the scutellum. *Wing* (Fig. 1C). Slightly smoky, the stigma brown yellowish, marginal maculae slightly brownish at apex of cells r and m; membrane microtrichose, except for extensive bare areas on basal half (cells c, sc, r1, dm and bm); tegula and basicosta black pilose, alula extensively bare medially, calypter whitish yellow, border whitish tawny, fringe yellow tawny, plumula yellow, halter white, knob white. *Legs* (Fig. 1C). Yellow to brown, pro and mesofemora brown, only slightly yellow on apical 1/3, respectively, metafemora brown, only slightly orange on apical 1/6, tibiae yellow with a dark ring near the middle, more prominent on the metalegs, tarsi brown, yellow pilose below, black pilose above. **Abdomen** (Fig. 1B). Elongate, black, with five pairs of lateral orange maculae reaching the apical 5/6 of the tergum, first tergum shining black, second to fourth tergum with a pair of broad subquadrate maculae, reaching the lateral margin in their full width; fifth tergum with a pair of small maculae in the basal corners. Pile orange on the sides basally, black down the middle and on the apical terga, as well as in the maculae; male genitalia as Fig. 2.

**Female.** (Fig. 1D–F). Similar to male except for normal sexual dimorphism. Abdominal maculae triangular and comparatively shorter than in the male, apically rounded, second to fifth tergum with maculae only reaching the apical 3/4, but not reaching the lateral margin in their full width. Frons shining above with a white pollinose transversal macula below. The female of *Argentinomyia andina* sp. nov. is similar in appearance to *Talahua fervida*, but *T. fervida* has a pair of small basolateral maculae on the sixth tergum and maculae on second to third tergum are longer than in *A. andina* sp. nov.

**Length** (*N* = 2). Body 11.2–11.5 mm; wing 10.8–11.1 mm.

##### Etymology.

The specific epithet *andina* (nominative, adjective feminine) is derived from the Andes South American mountain chain system where the type specimens were collected.

##### Distribution.

*Argentinomyia andina* sp. nov. (*N* = 8) is distributed on the west slope of the Central Cordillera in Northern Colombian Andes (Antioquia) at elevations between 1800–2700 m. a.s.l., in the provinces of Cauca (Fig. 15).

##### Type material.

**Holotype.** COLOMBIA ♂, Antioquia, Sonsón, Norí. Original label: “Colombia, Antioquia, Sonsón, vereda Norí / Norí mountain hill, Forest; 5°48.580'N, 75°16.142'E, 2896 m / 1-12.iv.2018, Malaise trap, Leg. A.L. Montoya and J.P. Carmona / CEUA 103430”. “HOLOTYPE / *Argentinomyia andina* / Montoya & Wolff 2020” [red, handwritten except first line]”. The holotype is in good condition and deposited at the CEUA, Medellín, Colombia. **Paratypes.** COLOMBIA • 1 ♂ same data as for holotype (CEUA) but differs on: Net, 2.vii.2018, Leg. J.P. Carmona, J. Sauceda, J. Vallejo (CEUA 103385); 2♂, same, except; 24.v-4.vi.2019, Leg. A.L. Montoya; J. Sauceda; M. Posada (CEUA 103552-53); 1♂, Antioquia, Santa Elena, Vereda El Placer, El Robledal, 6°13.717'N, 75°30.267'E, 2480 m a.s.l., Van Sommeren-Rydon trap baited with fish, 1–5.iii.2007, Leg A. Vélez (CEUA 103551); 1♀, Antioquia, San José de la Montaña, Vereda El Congo, Sector La Laguna, 6°45.827'N, 75°42.104'E, 3100-3200 m a.s.l., Páramo, Van Sommeren-Rydon trap baited with fish, 10-11.ix.2011, Leg. L. Rios (CEUA 69016); 1♀, same, except; 46.013'N, 75°41.979'E, 3100–3183 m a.s.l., Malaise, 4–14.ii.2017, Leg. C. Henao; A. F. Sepúlveda (CEUA 103635); 1♂, Sonsón, Vereda San Francisco, Las Palomas A Mountain hill, 5°43.606'N, 75°15.371'E, 2749 m a.s.l., Forest, Net, 1-12.iv.2018, Leg. A.L. Montoya, J. Carmona (CEUA 103434).

##### Comments.

*Argentinomyia andina* sp. nov. inhabits pristine Andean forest and Páramo ecosystems in Colombia, being particularly abundant in forest.

#### Argentinomyia choachi

Taxon classificationAnimaliaDipteraSyrphidae

Montoya sp. nov.

4D4BA30E-66C1-524A-A24C-B78C1E5FBFEA

http://zoobank.org/AACD240E-CB96-4DEF-A2A5-BB5AB779EF79

Figure 3 , 15

##### Differential diagnosis.

Abdomen with a pair of large quadrangular maculae on the third and fourth tergum, sometimes slightly touching toward the middle. Legs black, metafemur only slightly yellow in the extreme apex. Coxae black. Metacoxa yellowish pilose. *Argentinomyia choachi* sp. nov. is similar in appearance to the female of *Argentinomyia andina* sp. nov. but in *A. andina* sp. nov. the third and fourth tergum have a pair of short rounded basal maculae; metafemur is extensively brown, only slightly orange on apical 1/6; all coxae yellow.

##### Type locality.

Colombia, department of Cundinamarca, Choachí municipality, La Victoria. 4°32.721'N, 73°55.884'E, 2450 m a.s.l.

##### Description.

**Figure 3.**
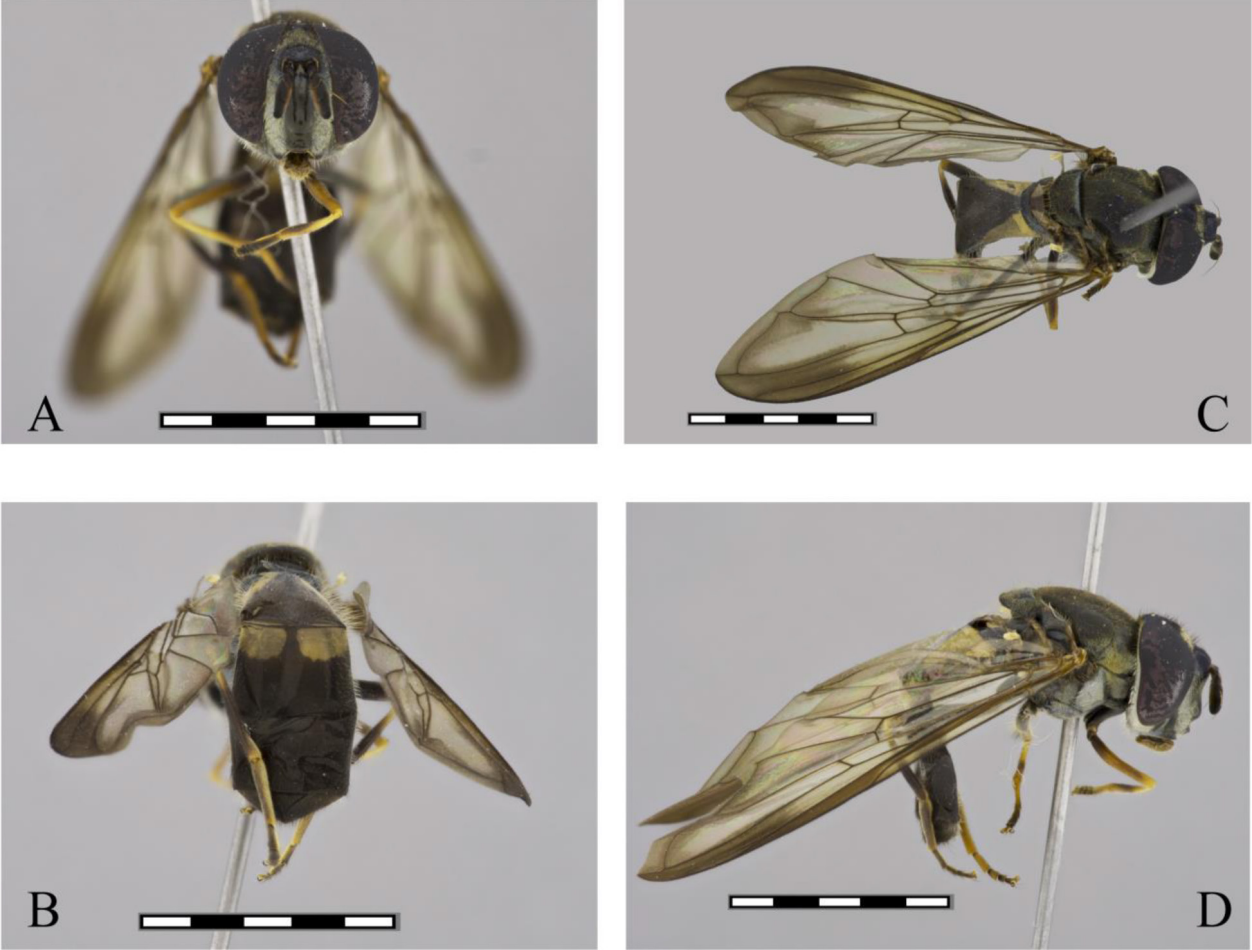
*Argentinomyia choachi* sp. nov., female (UNAB 5156): **A** head, frontal, female **B** posterior view **C** dorsal view **D** lateral view. Scale bars: 5 mm.

**Female. Head** (Fig. 3A, C). Black, covered with white pollinosity, oral tips, ocellar triangle, and a large triangular macula on the front, white pilose, pile on front black, on gena and face white, on ocellar triangle black, on the occiput white except for the dorsal pile, which are black. Antennae brown, orange-red ventrally, oval, longer than wide pedicel and the lower basal corner of basoflagellomere, ratio 1.0:1.3:3.1, arista orange, brown toward the tip. **Thorax** (Fig. 3C). Black cyaneous, the scutum shining, with iridescent to coppery reflections in the notopleura, with two median yellowish pollinose vittae on anterior half. *Wing* (Fig. 3C). Smoky apically, the stigma yellow-brown, membrane microtrichose, except for extensive bare areas on basal half (cells c, sc, r1, dm and bm); tegula black pilose, basicosta yellow pilose, alula bare, calypter whitish tawny, border whitish brown, fringe tawny, plumula yellow, halter white, knob white. *Legs* (Fig. 3C, D). Black, pro and mesofemora yellow on the apical two-thirds; metafemora only slightly yellow in the extreme apex, pro and mesotibia with lateral maculae on posterior medial edge, metatibia extensively brown, only yellow on basal 2/7 and apical 1/7; protarsi black, meso- and metabasitarsus yellow, the pile yellow, black on the metatibia and above on the tarsi; coxa black, metacoxa yellowish pilose. **Abdomen** (Fig. 3B). Elongate, black, with two pairs of lateral yellow pale maculae, first tergum shining black, second with broad lateral yellow maculae and only the apex black, third with rounded lateral maculae. Pile yellow-white on the maculae and only a few black down the middle and on the apical terga. Sterna black, black pilose.

**Male.** Unknown.

**Length** (*N* = 1). Body, 11.5–12.3 mm, wing, 10.2–10.4 mm.

##### Etymology.

The specific epithet “*choachi*” is a noun in apposition and refers to the name of the town where the type specimen was collected. *Coachí* is a Muiscas word derived from “*Ch-igua-chía*”, which means the window where the moon peeked, received the poetic name of “Window of the moon” (according to Miguel Triana). The Muiscas were indigenous people who inhabit the “Altiplano Cundiboyacense” formed by high plains on the eastern Cordillera in Colombian Andes between the departments of Cundinamarca and Boyacá, where the species was collected.

##### Distribution.

*Argentinomyia choachi* sp. nov. (*N* = 1) is present on the western slope of Oriental Cordillera in Colombia (Cundinamarca) at 2240 m. a.s.l., inhabiting cloud forest in the provinces of North Andean Páramo (Fig. 15).

##### Type material.

**Holotype.** COLOMBIA ♀, Cundinamarca, Choachí, La Victoria. Original label: “Colombia, Cundinamarca, Choachí, Vereda La Victoria / 4°32.721'N, 73°55.884'E, 2450 m a.s.l., Net / 18.iv.2011, Leg. J. Pérez (UNAB 5156)”. “HOLOTYPE / *Argentinomyia choachi* / Montoya 2020” [red, handwritten except first line]”. The holotype is in good condition and deposited at the UNAB.

##### Comments.

Only type specimen is known.

#### Argentinomyia huitepecensis

Taxon classificationAnimaliaDipteraSyrphidae

Montoya sp. nov.

0B2B58CB-3878-5A59-8296-9E9F3189C914

http://zoobank.org/0C676DC1-EE48-49D1-A770-A0778E4FAD40

Figures 4 , 5 , 16

##### Differential diagnosis.

Second tergum with a broad macula reaching apical 1/2. Third tergum with a short rounded basal fascia. Metacoxa black pilose. *Argentinomyia huitepecensis* sp. nov. is similar to *A. puntarena* sp. nov., but differs from it by having the antenna oval, longer than wide, orange ventrally; alula and costal cell bare; pro- and mesofemur yellow; metafemur orange on basal 1/5 and apical 3/5; pro, meso- and metabasitarsomere I–II yellow. *Argentinomyia huitepecensis* sp. nov. is also similar to *A. talamanca* sp. nov. a species with the antenna brown; alula and costal cell extensively microtrichose; femur extensively brown; the second tergum with a pair of small maculae on basal 1/5 (see ‘diagnostic features’ under each species or key).

##### Type locality.

México, department of Chiapas, San Cristóbal municipality, L.C. Huitepec 16°41.252'N, 92°35.979'E, 2520 m a.s.l.

##### Description.

**Figure 4.**
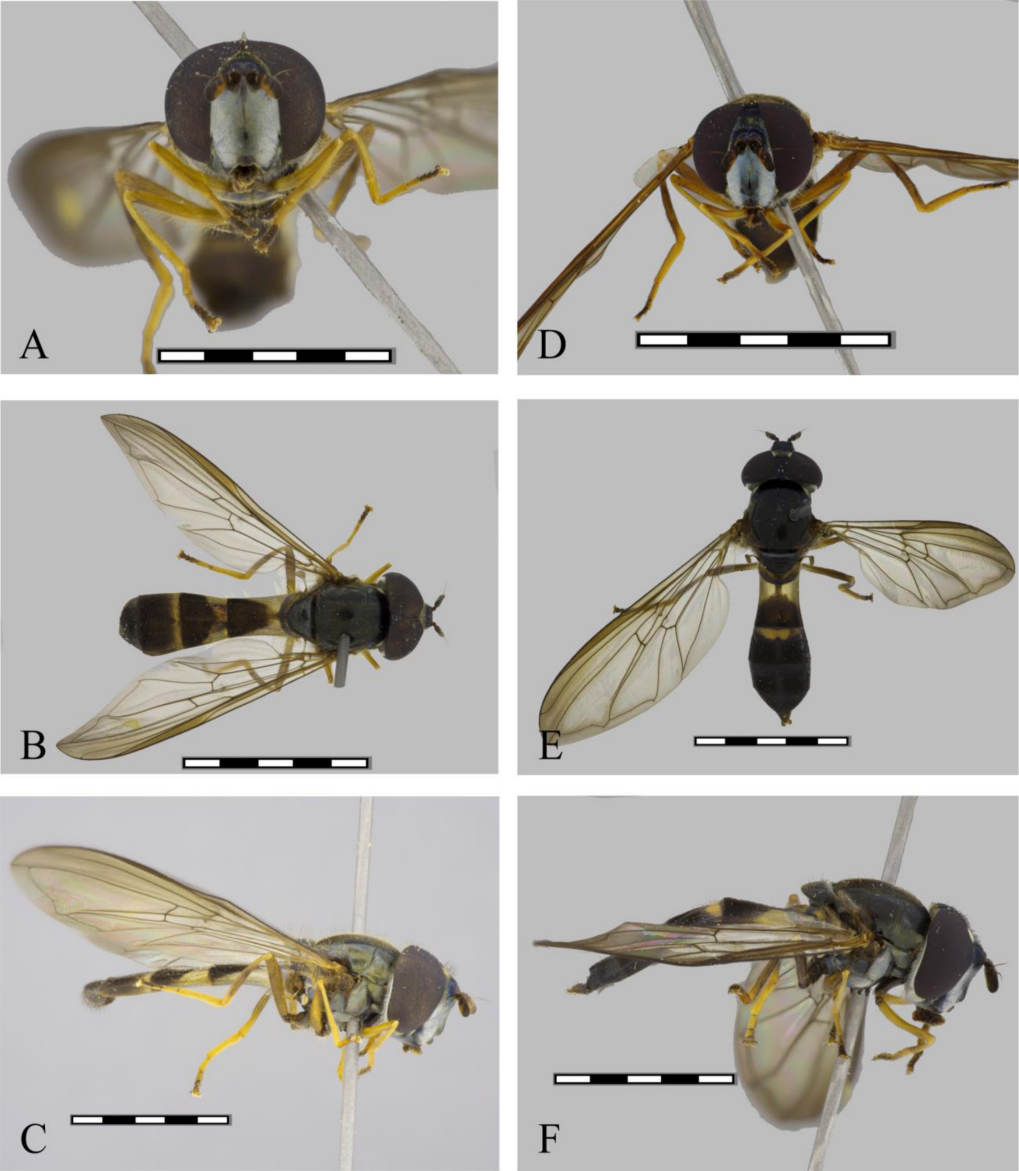
*Argentinomyia huitepecensis* sp. nov., male (ECOSCE 4925): **A** head, frontal, male **B** dorsal view **C** lateral view. Female (ECOSCE 24472): **D** head, frontal view **E** dorsal view **F** lateral view. Scale bars: 5 mm.

**Figure 5.**
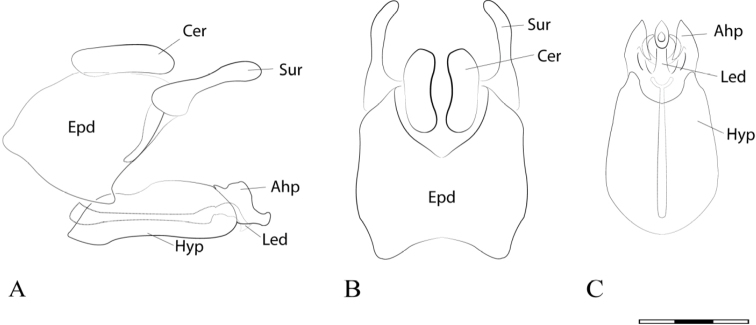
*Argentinomyia huitepecensis* sp. nov., male genitalia: **A** whole genitalia including epandrium, cercus, and surstylus, lateral view **B** epandrium, dorsal view **C** hypandrium, ventral view. Scale bar: 0.05 mm.

**Male. Head** (Fig. 4A, C). Black, covered with white pollinosity, oral tips, ocellar triangle, and a large triangular macula on the front, brown pilose, pile on front black, on gena and face white pilose, on ocellar triangle black pilose, on the occiput white except the dorsal pile, which are black, frontal triangle golden metallic. Antennae brown, orange-red ventrally, oval, longer than wide pedicel and the lower basal corner of basoflagellomere, long, ratio 1.0:1.2:3.0, arista orange, dark brown toward the tip. **Thorax** (Fig. 4C). Black, the scutum opaque, shining, with iridescent to opaque reflections, with two median brownish pollinose vittae on anterior half. *Wing* (Fig. 4C). Slightly smoky, the stigma brown, membrane microtrichose, except for extensive bare areas on basal half (cells, c, sc, r1, dm and bm); tegula black pilose, basicosta yellow pilose, alula bare, calypter whitish, border whitish, fringe yellow, plumula yellow, halter yellow, knob white. *Legs* (Fig. 4C). Yellow, pro- and mesofemur yellow; metafemur orange on basal 1/5 and apical 3/5; pro- and mesotibia orange with a brown macula on posterior medial edge, metatibia extensively brown, only orange brownish on basal 2/3; metacoxa black pilose; pro, meso- and metabasitarsomere I-II yellow, the pile yellow, black on the metatibia and above on the tarsi. **Abdomen** (Fig. 4B). Elongate, black, first tergum shining black, second tergum with a pair of broad maculae, reaching apical 1/2, third tergum with a short rounded basomedial fascia. Pile orange on the sides basally, black down the middle and on the apical terga, as well as in the maculae. Male genitalia as Fig. 5.

**Female.** (Fig. 4D–F). Similar to male except for normal sexual dimorphism. Abdominal maculae on tergum third comparatively shorter than in the male and restricted to the center of the tergum, no reaching the lateral margin, maculae on fourth and fifth tergum absent.

**Length** (*N* = 5). Body 12.5–12.8 mm; wing 11.4–11.7 mm.

##### Etymology.

The specific epithet *huitepecensis* (noun in the genitive case) is derived from the Mixtec (native language spoken in México) word “*Huitztli*” which means: thorns, “*Tépeltque*” means: hill, combined with the Latin suffix -*ensis*, meaning from a place. The name is given in reference to the Huitepec Ecological Reserve where the species was collected.

##### Distribution.

*Argentinomyia huitepecensis* sp. nov. (*N* = 7) is the northernmost distributed species of the larger *Argentinomyia*, recorded on the western slope of the Chiapas-Guatemalan Highlands, and inhabiting cloud forest at an elevation between 1800 to 2400 m. a.s.l. The species is exclusively known from the province of Chiapas Highlands (Fig. 16).

##### Type material.

**Holotype.** MÉXICO ♂, Chiapas, San Cristóbal, L.C. Huitepec. Original label: “Mexico, Chiapas, San Cristóbal, L.C. Huitepec / 16°41.252'N, 92°35.979'E, 2520 m a.s.l., 5.x.2010 / wpt 12, P. Sagot, no 6, collect #4301, ECO-SC-E 4925”. “HOLOTYPE / *Argentinomyia huitepecensis* / Montoya 2020” [red, handwritten except the first line]”. The holotype is in good condition and deposited at the ECO-SC-E, Chiapas, México. “Identified as *Argentinomyia* sp. 16 by P. Sagot”. **Paratypes.** MÉXICO • 1 ♂ same data as for holotype (ECO-SC-E) but differs on: 16°41.255'N, 92°35.979'E, 2450 m a.s.l., 20.xi.2009, wpt 30, P. Sagot, no 16, collect #2504 (ECO-SC-E 24471), sp. 16; 1♂, 2450 m a.s.l., 13.i.2010, wpt 25, P. Sagot, no 8, collect #2950. 1♀, 2400 m a.s.l., 20.xi.2009. wpt 30, no 16, collect #2504 (ECO-SC-E 24472); 1♂, 12.x.2010, wpt 13, P. Sagot, no 48, collect #2504 (ECO-SC-E 24471), sp. 16; 1♂, 2560 m a.s.l., 12.x.2010, wpt 13, P. Sagot, no 48, collect #4413 (ECO-SC-E), sp. 16; 1♂, 2310 m a.s.l., 4.i.2011, wpt 34, P. Sagot, collect #4925 (ECO-SC-E), sp. 10; 1♀, 2390 m a.s.l., 31.x.2010, wpt 9, P. Sagot, no 6, collect #4539 (ECO-SC-E) sp. 10.

#### Argentinomyia puntarena

Taxon classificationAnimaliaDipteraSyrphidae

Montoya sp. nov.

37DC0BED-A66F-54A0-B493-415499ACEEC2

http://zoobank.org/67E5B850-AC47-4027-B412-B1CDDA2785AB

Figures 6 , 7 , 16

##### Differential diagnosis.

Antenna orange ventrally, oval, longer than wide. Costal cell hyaline, bare basally. Alula bare medially. Pro- and mesofemur yellow. Metafemur orange on basal 1/5 and apical 3/5. Probasitarsomere, meso- and metabasitarsomere I–II yellow. Second tergum with a broad macula reaching apical 1/3. Third tergum with a broad macula, short in fourth. Sternite fourth to fifth black pilose. *Argentinomyia puntarena* sp. nov. is similar in appearance to *Argentinomyia talamanca* sp. nov., but in *A. talamanca* sp. nov. the antenna is brown; alula and costal cell are extensively microtrichose; femur is extensively brown; the second tergum has a pair of small maculae on basal 1/5 (see ‘diagnostic features’ under each species or key).

##### Type locality.

Costa Rica, department of Puntarenas, Coto Brus municipality, Sendero entre Estación Tres Colinas y Laguna Seca, 9°1.279'N, 82°, 50.337'E, 2100–2550 m a.s.l.

##### Description.

**Figure 6.**
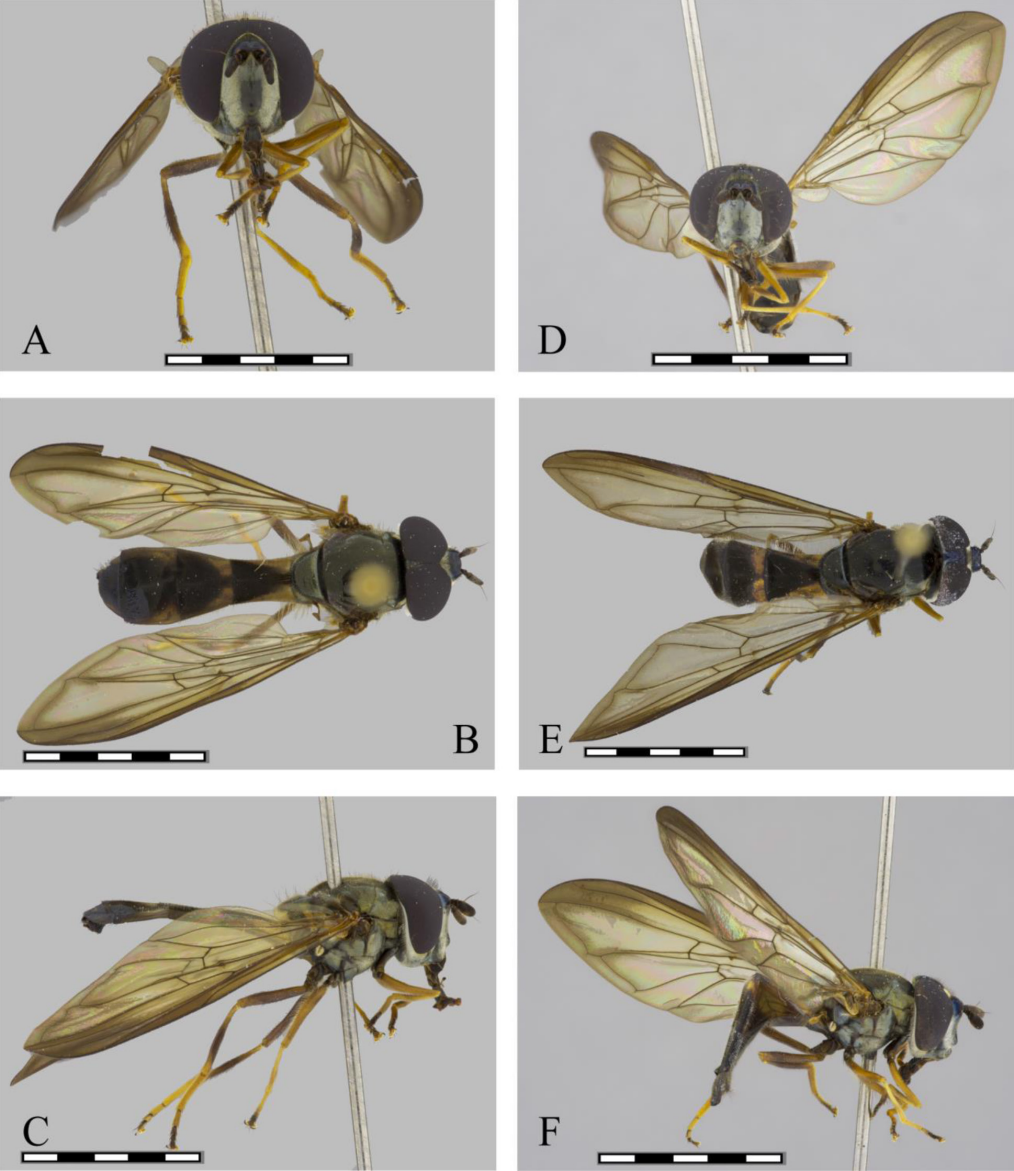
*Argentinomyia puntarena* sp. nov., male (InBio CRI000311623): **A** head, frontal, male **B** dorsal view **C** lateral view. Female (InBio CRI002427774): **D** head, frontal view **E** dorsal view **F** lateral view. Scale bars: 5 mm.

**Figure 7.**
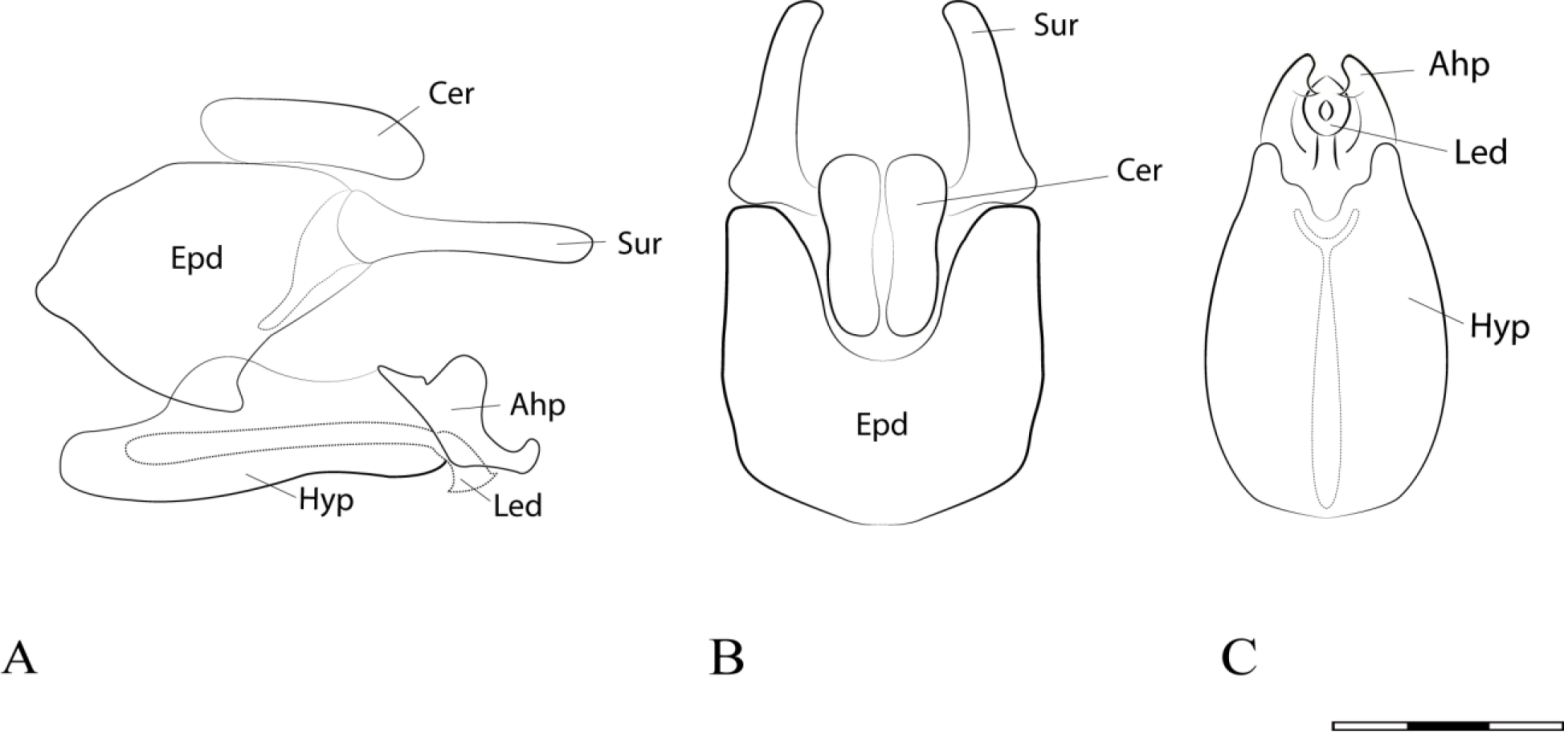
*Argentinomyia puntarena* sp. nov., male genitalia: **A** whole genitalia including epandrium, cercus, and surstylus, lateral view **B** epandrium, dorsal view **C** hypandrium, ventral view. Scale bar: 0.05 mm.

**Male. Head** (Fig. 6A, C). Black, covered with white pollinosity, ocellar triangle, and a large triangular macula on the front, brown pilose, pile on front black, on gena and face white pilose, on ocellar triangle black pilose, on the occiput white except for the dorsal pile, which are black, frontal triangle golden metallic. Antennae brown, orange-red ventrally, oval, longer than wide pedicel and the lower basal corner of basoflagellomere, ratio 1.0:1.1:2.9, arista orange, dark brown toward the tip. **Thorax** (Fig. 6C). Black, scutum shining, with iridescent to opaque reflections, with two median brownish pollinose vittae on anterior half. *Wing* (Fig. 6C). Slightly smoky, the stigma brownish, membrane microtrichose, except for extensive bare areas on basal half (cells, sc, r1, dm and bm); costal cell hyaline, bare on basal 1/2; marginal maculae restricted to surrounding areas of veins R1, R4+5 and M1 apically; tegula black pilose, basicosta yellow pilose, alula bare medially, calypter whitish, border whitish, fringe yellow, plumula yellow, halter yellow, knob white. *Legs* (Fig. 6C). Yellow, pro- and mesofemur yellow; metafemur brown, except orange on basal 1/5 and apical 1/5; pro and mesotibia orange, metatibia extensively brown, only orange brownish on basal 2/3; probasitarsomere, meso- and metabasitarsomere I–II yellow, the pile yellow, black on the metatibia and above on the tarsi. **Abdomen** (Fig. 6B). Elongate, black, the first tergum shining black, orange laterally, the second tergum with a broad macula reaching apical 1/3 laterally; third tergum with a broad triangular macula, which is short in the fourth tergum; fourth and fifth sterna black pilose. Pile orange on the sides basally, as well as in the maculae, black down the middle and on the terga apex. Male genitalia as Fig. 7.

**Female.** (Fig. 6D–F). Similar to male except for normal sexual dimorphism. Frons with pollinose transversal maculae below. Abdominal maculae are comparatively shorter than in the male and indistinguishable in the fourth tergum.

**Length** (*N* = 4). Body 12.4–12.6 mm; wing 11.3–11.5 mm.

##### Etymology.

The specific epithet *puntarena* is a noun in apposition and refers to the province where the type series was collected.

##### Distribution.

*Argentinomyia puntarena* sp. nov. (*N* = 5) is distributed through the west slope of the Talamanca Cordillera in Costa Rica (Puntarenas, San José) at an elevation between 1000 to 2550 m. a.s.l., in the province of Puntarena-Chiriquí (Fig. 16). *Argentinomyia puntarena* sp. nov. occurs in sympatry with *A. talamanca* sp. nov. in the Puntarena-Chiriquí province.

##### Type material.

**Holotype.** COSTA RICA ♂, Puntarenas, Coto Brus, Sendero entre Estación Tres Colinas y Laguna Seca. “Original label: “Costa Rica: Puntarenas, Coto Brus, Sendero entre / Estación Tres Colinas y Laguna Seca / 9°1.279'N, 82°50.337'E (L.S. 344300_565800), 2100–2550 m a.s.l. /24.vii.2000, Manual, A. Picado Leg., #59166 (INBio 000311623)”. “HOLOTYPE / *Argentinomyia huitepecensis* / Montoya 2020” [red, handwritten except the first line]”. The holotype is in good condition and deposited at the INBio museum, in Costa Rica. “identified as *Argentinomyia* sp. 16 by Thompson”. **Paratypes.** COSTA RICA • 1 ♂, Puntarenas, Monteverde, San Luis, 10°16.644'N, 84°47.271'E (L.S. 250850_449250), 1000-1350 m a.s.l., 7.iv.1995, Fuentes #4801 (INBio CRI002202643); 1♀, San José, San Gerardo de Dota, Sevegre Lodge near Rio Sevegre, 9°33.000'N, 83°48.000'E (L.S. 387400_482700), 2200 m a.s.l., 18–21.viii.1995, A.L. Norrbom (USNM ENT 00036925); 1♀, same data as for preceding, 2000–2500 m a.s.l., 22.ii.1992, Tachinidae and Syrphidae course (INBio CRI000406820); 1♀, Farm Zacatales, 2100 m a.s.l., 8–10.viii.1995, M.A. Zumbado, #6280 (INBio CRI002427774).

##### Comments.

*Argentinomyia puntarena* sp. nov. and *Argentinomyia talamanca* sp. nov. can be confused or mistakenly identified as *Xanthandrus mexicanus* Curran, 1930 due to the superficial similarity of these species. However, *X. mexicanus* can be distinguished by the presence of the antennal cavity broadly confluent (synapomorphy for *Xanthandrus*); the central portion of epistoma moderately prominent; the face entirely white pollinose and pilose; the pleura black with white pollinosity; metaepisternum with some fine subappressed long pile (distinctive of *Xanthandrus*); the abdomen oval, wide and flat, opaque, with yellow-orange triangular maculae on second to the fourth tergum, and the male genitalia with surstylus elongated, apically widened (Borges and Pamplona 2003:162, figs 33–37).

#### Argentinomyia quimbaya

Taxon classificationAnimaliaDipteraSyrphidae

Montoya & Wolff sp. nov.

B4EB641B-605A-51D2-ADC1-CE8F391FA4EB

http://zoobank.org/242B50C6-57FD-4B8E-AA43-65337E1A3D7D

Figures 8 , 9 , 15

##### Differential diagnosis.

Legs extensively yellow. Tegula yellow pilose and the halter entirely yellow. Abdomen with four pairs of lateral broad yellow maculae. *Argentinomyia quimbaya* sp. nov. is similar in appearance to *Argentinomyia andina* sp. nov., but in *A. andina* sp. nov. the legs are extensively brown, metafemur only slightly orange on apical 1/6. Tibiae yellow with a dark ring near the middle. Fifth tergum with a pair of small maculae in the basal corners (see ‘diagnostic features’ under each species or key).

##### Type locality.

Colombia, department of Caldas, Manizales municipality, Corregimiento Las Palmas, Parque Rio Blanco, 5°5.017'N, 75°25.133'E, 2782 m a.s.l.

##### Description.

**Figure 8.**
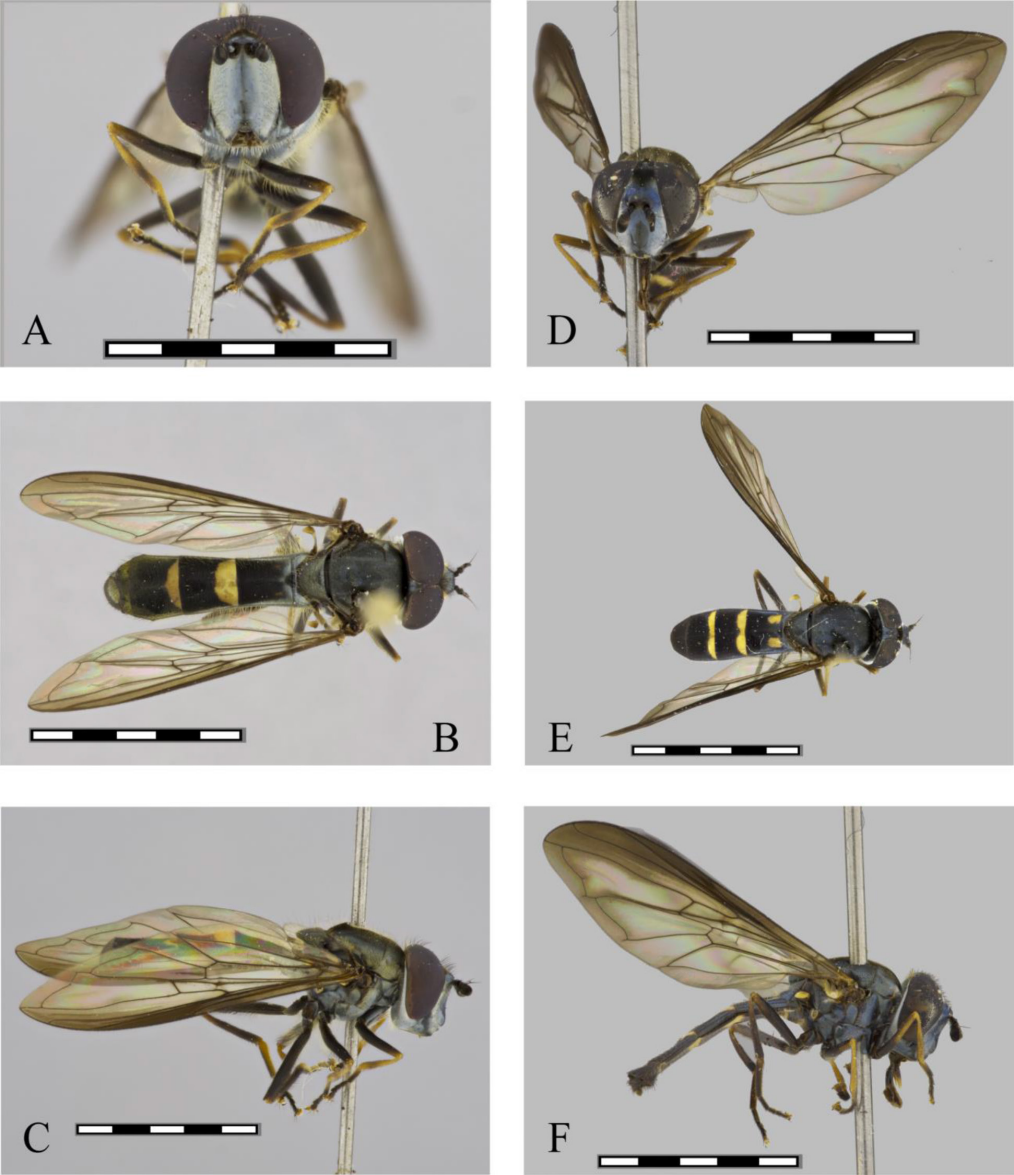
*Argentinomyia quimbaya* sp. nov., male (USMN ENT 000035733): **A** head, frontal, male **B** dorsal view **C** lateral view. Female, Paratype (CEUA 87109): **D** head, frontal view **E** dorsal view **F** lateral view. Scale bars: 5 mm.

**Figure 9.**
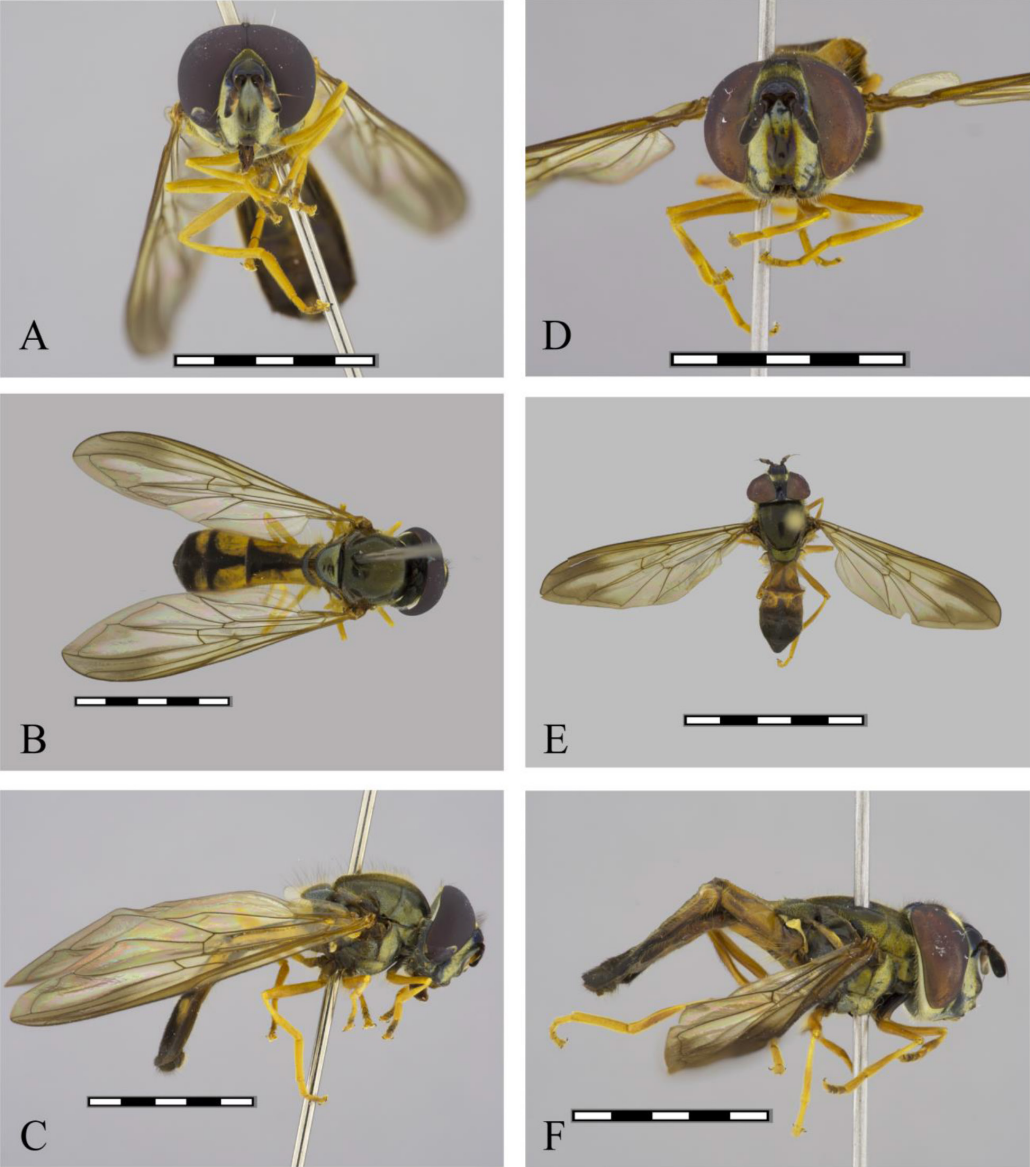
*Argentinomyia quimbaya* sp. nov., male genitalia: **A** whole genitalia including epandrium, cercus, and surstylus, lateral view **B** epandrium, dorsal view **C** hypandrium, ventral view. Scale bar: 0.05 mm.

**Male. Head** (Fig. 8A, C). Black, covered with yellowish gray pollinosity, shining black on the prominent round tubercle, oral tips, ocellar triangle, and a large triangular macula on the front; pile on front black, on gena and face yellow, on ocellar triangle black, on the occiput yellow except the dorsal pile, which are black. Antennae brown, orange-red ventrally, oval, longer than wide pedicel and the lower basal corner of basoflagellomere, long, ratio 1.0:1.2:3.1, arista orange, dark brown toward the tip. **Thorax** (Fig. 8C). Black, the scutum shining, covered with coppery pollen and short golden pile with many long black hairs that appear yellowish at the base; these hairs become longer posteriorly and longer on the scutellum, fringe of scutellum yellow; pleura yellow pollinose and pilose. *Wing* (Fig. 8C). Smoky; the stigma brown yellowish; membrane microtrichose, except for extensive bare areas on basal half (cells c, sc, r1, dm, and bm); tegula yellow pilose, basicosta yellow pilose, alula extensively bare, calypter and plumule yellow; halter entirely yellow. *Legs* (Fig. 8B). Yellow, protarsi 1 yellow, 2–4 brown, 5 yellow, mesotarsi 1–2 yellow, 3–4 black, 5 yellow, metatarsi yellow. **Abdomen** (Fig. 8B). Elongate, black, with four pairs of lateral yellow maculae; first tergum shining, laterally yellow, second tergum with the broad lateral yellow maculae reaching the segment apex, third tergum with wide rectangular lateral maculae, which are apically rounded and reach the apical 6/7 of the segment, fourth tergum with still wider but less elongate maculae, reaching only ½ of the segment length. Pile yellow on the sides basally, black down the middle and on the apical terga. Male genitalia as Fig. 9.

**Female.** (Fig. 8D–F). Similar to male except for normal sexual dimorphism. Abdominal maculae are comparatively wider than in the male. Front narrow above, not the much wider than the ocellar triangle, shining above with pollinose transversal maculae below; legs extensively yellow.

**Length** (*N* = 2). Body 11.8–12.3 mm; wing 11.5–11.9 mm.

##### Etymology.

The specific epithet *quimbaya* (noun in the genitive case) refers to the indigenous people who inhabit the Central Cordillera of the Colombian Andes in pre-Colombian times, between the departments of Caldas and Risaralda. The name also refers to the Flora and Fauna Sanctuary (SFF, acronym in Spanish) Otún Quimbaya, where part of the type series was collected.

##### Distribution.

*Argentinomyia quimbaya* sp. nov. (*N* = 2) is distributed on the western slope of the Central Cordillera of Colombia (in two very neighbouring Andean states, Caldas and Risaralda) at an elevation between 2700 to 2782 m. a.s.l. in the provinces of Cauca (Fig. 15).

##### Type material.

**Holotype.** COLOMBIA ♂, Colombia, Caldas, Manizales, Corregimiento Las Palmas, Parque Rio Blanco. Original label: “Colombia, Caldas, Manizales, Corregimiento Las Palmas / Parque Rio Blanco, 5°5.017'N, 75°25.133'E, 2782 m a.s.l. / Net, 18.ii.2006, Leg. B.J. and F.C. Thompson (USMN ENT 000035733)”. “HOLOTYPE / *Argentinomyia quimbaya* / Montoya & Wolff 2019 [red, handwritten except first line]”. The holotype is in good condition and deposited at the USMN in Washington D.C., USA. **Paratype.** COLOMBIA • 1 ♀, Risaralda, Otún Quimbaya, Peña Bonita, El Jordán 4°44.617'N, 75°31.494'E, 2640–2800 m a.s.l., Van Sommeren-Rydon- Chicken entrails, 13–14.iv.2011, N. Uribe (CEUA 87109).

#### Argentinomyia talamanca

Taxon classificationAnimaliaDipteraSyrphidae

Thompson sp. nov.

79A5F8AE-E7AF-5145-8298-DA1C810A24F7

http://zoobank.org/68AFF494-F5E9-4A1F-8C0D-54E523778B2B

Figure 10 , 11 , 16

##### Differential diagnosis.

Antenna brown, rounded, as long as wide; face opaque, white pollinose and pilose. Wing, alula and, costa cell extensively microtrichose, except for extensive bare areas on basal half (cells, sc, r1, dm, and bm); costal cell brownish, calypter and border whitish, fringe plumula and, halter yellow, knob white. Femora and tarsi extensively brown. Abdomen, second tergum with a pair of small narrow maculae reaching basal 1/5; third and fourth tergum with basomedial maculae, reaching 2/5 and 1/5, respectively; fifth sternite black pilose. *Argentinomyia talamanca* sp. nov. is similar in appearance to *Argentinomyia puntarena* sp. nov., but in *A. puntarena* sp. nov. the antenna is orange ventrally, oval, longer than broad; costal cell hyaline, bare basally; alula bare medially; pro- and mesofemur yellow; metafemur orange on basal 1/5 and apical 3/5; pro-, meso- and metabasitarsomere I–II yellow; second tergum with a broad maculae reaching the apical 1/3; third tergum with a broad macula, short in the fourth; sternite fourth to fifth black pilose (see ‘diagnostic features’ under each species or key).

##### Type locality.

Costa Rica, department of Cartago, Rio Macho muncipality, Estación Ojo de Agua, 9°36'2.23"N, 83°45'43.31"E, 3000 m a.s.l.

##### Description.

**Figure 10.**
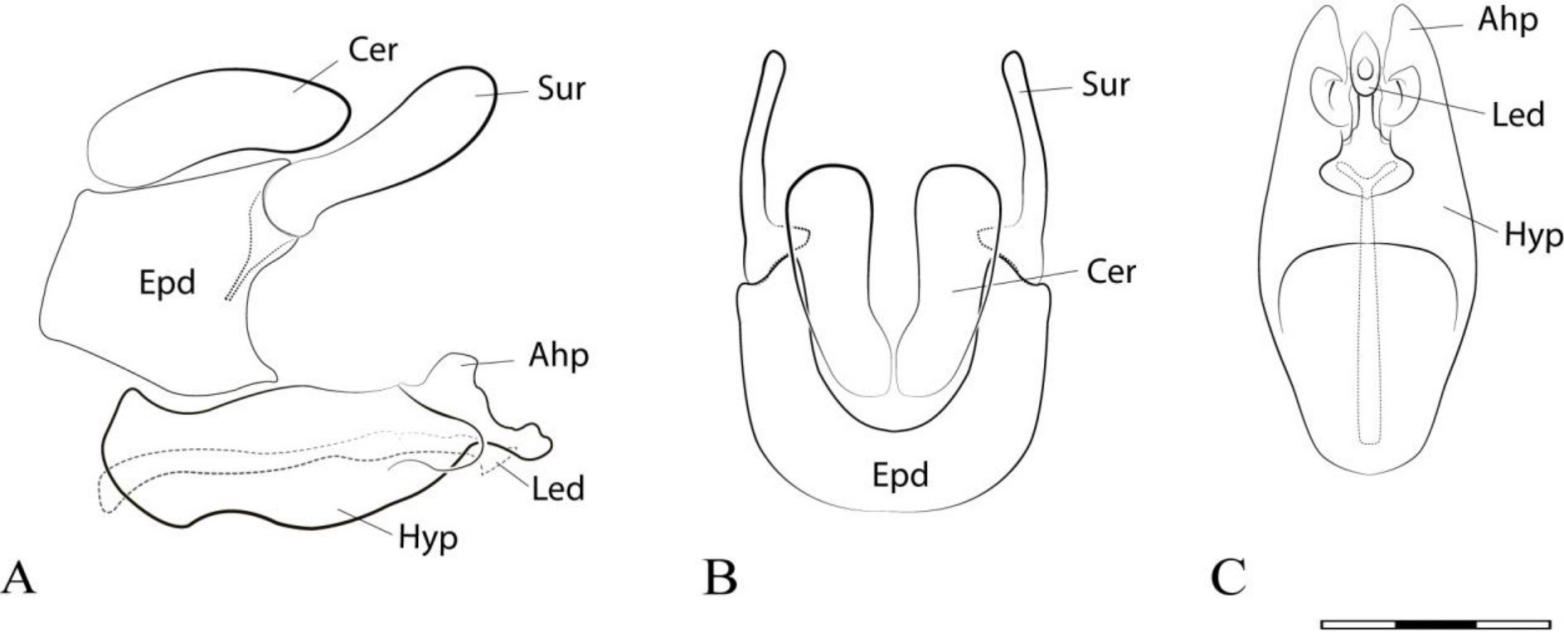
*Argentinomyia talamanca* sp. nov., male (USNM ENT 00036926): **A** head, frontal, male **B** dorsal view **C** lateral view. Female (INBio CRI002462383): **D** head, frontal view **E** dorsal view **F** lateral view. Scale bars: 5 mm.

**Figure 11.**
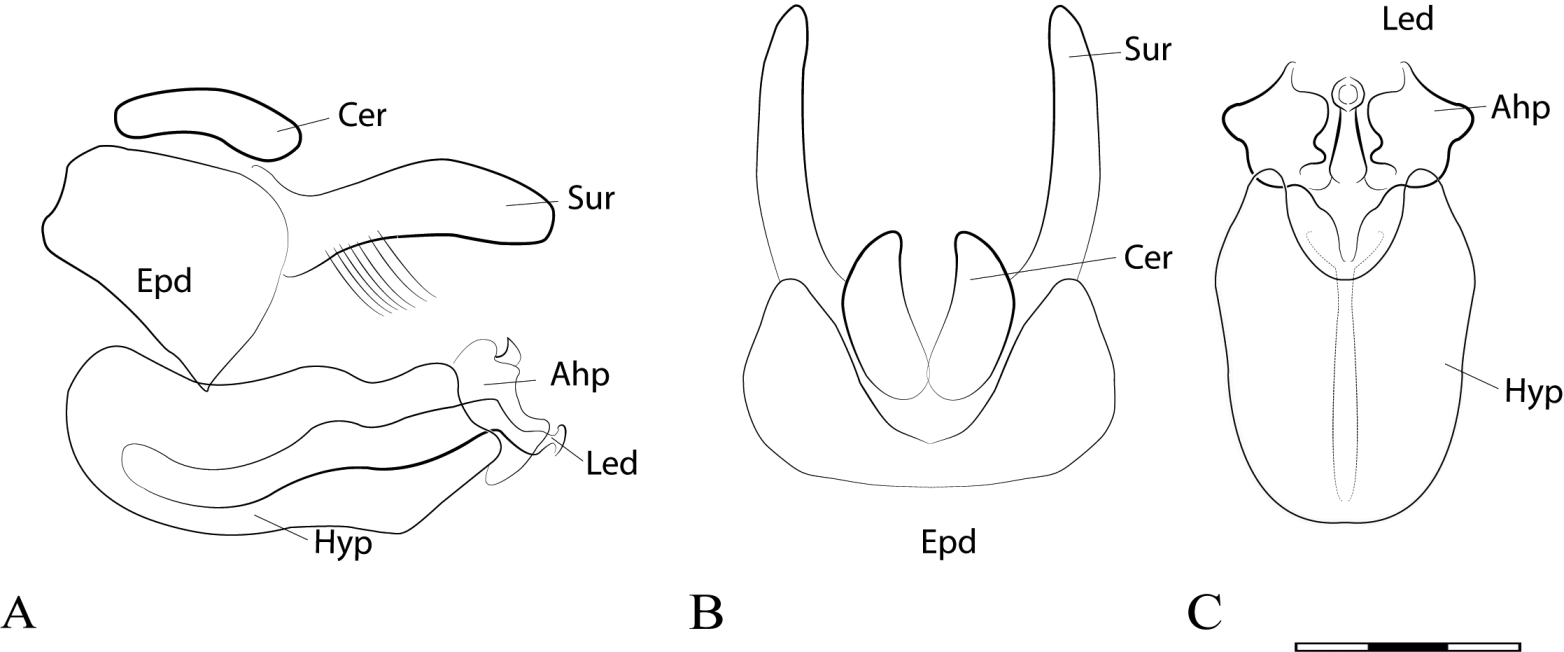
*Argentinomyia talamanca* sp. nov., male genitalia: **A** whole genitalia including epandrium, cercus, and surstylus, lateral view **B** epandrium, dorsal view **C** hypandrium, ventral view. Scale bar: 0.05 mm.

**Male. Head** (Fig. 10A, C). Black, covered with white pollinosity, oral tips, ocellar triangle, and a large triangular macula on the front, brown pilose, pile on front black, on gena and face white pilose, on ocellar triangle black pilose, on the occiput white except the dorsal pile, which are black, frontal triangle golden metallic; face with a carina above the tubercle. Antennae brown, rounded, as long as wide, ratio 1.1:1.2:2; arista orange, dark brown toward the tip. **Thorax** (Fig. 10B, C). Black, the scutum shining, with iridescent to opaque reflections, with two median brownish pollinose vittae on anterior half. **Wing** (Fig. 10D). Slightly smoky, the stigma brown, membrane extensively microtrichose, except for extensive bare areas on basal half (cells, sc, r1, dm, and bm); costal cell brownish, extensively microtrichose; tegula black pilose, basicosta yellow pilose, alula extensively microtrichose; calypter whitish, border whitish, fringe yellow, plumula yellow, halter yellow, knob white. **Legs** (Fig. 10C). Dark brown, pro- and mesofemur brown, orange on apical 1/8; metafemur extensively brown; pro and mesotibiae orange with a brown macula on posterior medial edge, metatibia extensively brown, only orange brownish on basal 2/3; tarsi brown, only meso-basitarsi orange basally, the pile yellow, black on the metatibia and above on the tarsi. **Abdomen** (Fig. 10B). Elongate, black, first tergum shining black, second with a pair of small narrow maculae, occupying 2/5 of segment length, third and fourth with basomedial maculae, reaching 2/5 and 1/5, respectively. Pile orange on the sides basally, black down the middle and on the apical terga, as well as in the maculae; sternite IV black pilose. Male genitalia as Fig. 11.

**Female.** (Fig. 10D–F). As male, except for usual sexual dimorphism and following differences: frons with a pollinose transversal macula below. Abdomen with a pair of square-like and apically rounded maculae on the second tergum, which occupies the anterior half of the tergum and comparatively larger than in the male. Profemur extensively yellow on anterior half. Female of *Argentinomyia talamanca* is similar in appearance to *Argentinomyia puntarena* sp. nov. but in *A. puntarena* the antenna is orange ventrally; alula and costal cell bare; pro, meso- and metabasitarsomere I–II yellow; second tergum with a lateral macula reaching the 5/6 of tergum length.

**Length** (*N* = 4). Body 10.5–11.4 mm; wing 9.2–9.8 mm.

##### Etymology.

The noun in apposition ‘*Talamanca*’ refers to the cordillera where the species was collected in Costa Rica.

##### Distribution.

*Argentinomyia talamanca* sp. nov. (*N* = 21) is distributed through the Talamanca Cordillera in Costa Rica (Cartago, Limón, Puntarenas, San José) at an elevation between 2400 to 3600 m. a.s.l. in the province of Puntarena-Chiriquí (Fig. 16).

##### Type material.

**Holotype.** COSTA RICA ♂, Cartago, Rio Macho, Estación Ojo de Agua. Original label: “Costa Rica, Cartago, Rio Macho, Estación Ojo de Agua, 9°36'2.23"N, 83°45'43.31"E, (L.S. 396700_482200), 3000 m a.s.l., 25.vii.1999, A. Pinto Leg., #62964 (INBio 003321800)”. “HOLOTYPE / *Argentinomyia talamanca* / Thompson 2020” [red, handwritten except first line]”. The holotype is in good condition and deposited at the INBio museum, in Costa Rica. “Identified as *Xanthandrus* 75-10 by Thompson 1971”. **Paratypes.** COSTA RICA• 1♂, Cartago, Rio Macho, Estación Ojo de Agua, A orillas de la carretera Interamericana, 9°36'2.23"N, 83°45'43.31"E, (L.S. 396700_482200), 3000 m a.s.l., 25.vi.1999, A. Pinto. Libre, #46825 (INBio 0003321799); same data except: 1♂, 26.vi.1997, B. Gamboa #62964, (INBio 0002566220); 1♀, #46825 (INBio 0002566223); 1♀, 9°35'5.48"N, 83°44'14.01"E, 2850 m a.s.l., 11.xii.1997, E. Alfaro, #48829 (INBio CRI002525323); 1♀, Sendero a Torre 47, 9°35'44.84"N, 83°44'37.94"E, 2960 m a.s.l., 26.iii.1997, A. Picado, #45541 (INBio CRI002537729); 1♂, Sendero a Torre 46, 2760 m a.s.l., 9°33'4.23"N, 83°43'45.13"E, 12.iv.1997, B. Gamboa, #46759 (INBio CRI002565331); 1♂, Cartago, Estación Cuericí, El mirador, 4km al E. Villa Mills, Subparamo, 9°31'6.97"N, 83°33'12.55"E, 2900 m a.s.l., 7.xii.1996, A. Picado, #45170 (Collector # 489, 900-093) (INBio CRI002462385); , 1♀, #45170 (INBio CRI002462383); 1♀, sendero Cerro Cuericí, Limite P.N. Chiripo, 9°31'24.05"N, 83°30'15.03"E (L.S. 396700_482200), 3050 m a.s.l., 5.i.1996, A. Picado, #6799 (INBio CRI002367564); 1♂, camino la Auxiliadora, 3.5km E de Villa Mills, 9°32'47.29"N, 83°42'31.97"E, 2700 m a.s.l., 8.vii.1996, A. Picado, #7721 (INBio CRI002467120); 1♂, Cartago, Cerro Urán, 9°30'5.70"N, 83°30'56.31"E, 3600 m a.s.l., 1.v.1997, A. Picado, #46214 (INBio CRI002504307); 1♂, Limón, La Amistad, Bratsi, Cerrito en Fila Dudu-Apri, 9°16'12.31"N, 83°2'53.69"E (L.S. 356400_566000), 3100 m a.s.l., 23.vi.2000, Manual, A. Picado, #59164 (INBio 000311588); 1♂, sendero Circular, 9°17'44.79"N, 83°2'35.59"E (L.S. 340258_577465), 2406 m a.s.l., 20.vi-5.vii.2003, Libre, D. Rubi, #74159 (INBio 0003724228); 1♂, #74159 (INBio 0003724233). Puntarenas, 5 km, S. Rincón, 15.iii.1973, E.W. Barrows (1♀); 1♀, San José, Barva, i.1997, FCT group (USNM ENT 00036927); 1♂, San José, Estación Cerro de la Muerte, Km 92, Carretera Interamericana, 9°34'22.79"N, 83°44'45.87"E (L.S. 390300_491700 #57449), 3140 m a.s.l., 24.iii.2000, M.A. Zumbado (INBio 0003168749); 1♂, San José, Cerro de la Muerte, 6 km, W. Villa Mills, Inter-Am. Hwy., 9°34'30.93"N, 83°37'47.69"E, 3340 m a.s.l., on flower of Compositae, 2.v.1972, E. R. Heithaus (Collector # 489, 900-093) (INBio 15810, dissected); on flower of Rosaceae, 25.viii.1971, E. R. Heithaus (Collector # 830) (1♀, INBio 4788, identified as *Xanthandrus* 75-10 Thompson); 1♂, San José, Los Santos, Camino a Providencia de Dota, 9°37'0.44"N, 83°50'19.14"E, 2900 m a.s.l., 18.i.1997, M. Segura, #45294 (INBio CRI002535232); 1♂, San José, PamAn Hwy, Km 89, Cerro de la Muerte, Las Torres, 9°34'N, 83°45'E, 3367 m a.s.l., 18-19.viii.1995, A.L. Norrbom (USNM ENT 00036926).

##### Comments.

*Argentinomyia talamanca* sp. nov. is only known from the Talamanca Cordillera in Costa Rica.

#### Genus Talahua

Taxon classificationAnimaliaDipteraSyrphidae

Fluke

EF8B5DC2-CDAC-50DB-AA57-BE90416EED65

Talahua Fluke, 1945: 22. Type species, Melanostoma fervidum Fluke, 1945 by original designation. Described as subgenus of Melanostoma.

##### Referens.

Fluke 1945: 23 (description, key), figs 34 (head), 35 (male genitalia), 53 (abdomen), Fluke 1957: 279, fig. 123 (male genitalia), Thompson et al. 1976: 45 (cat.), Thompson 1999: 325–338 (key and taxonomic notes), Montoya et al. 2012 (distributional records), Huo 2014 (diagnosis), Thompson and Skevington 2014 (key and review of melanostomine genera groups), Montoya 2016: 463 (cat.), Marín-Armijos et al. 2017: 184 (distributional records).

#### Talahua fervida

Taxon classificationAnimaliaDipteraSyrphidae

Fluke, 1945

050D55C5-C91A-5E80-AD3B-8621FF225B78

**Figure 12.**
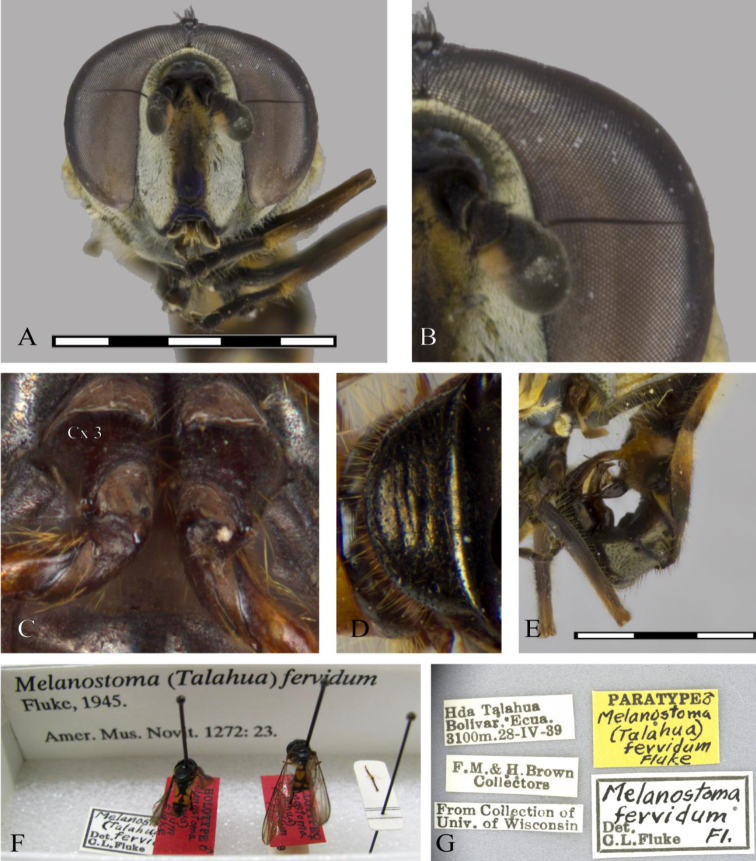
Genus *Talahua* Fluke: **A** head, frontal, male **B** basoflagellomere, frontal view **C** metacoxa pile tuft, ventral view **D** scutellum emarginated, dorsal view **E** male genitalia, lateral view **F** holotype, AMNH **G** paratype label, USNM. Scale bars: 5 mm.

**Figure 13.**
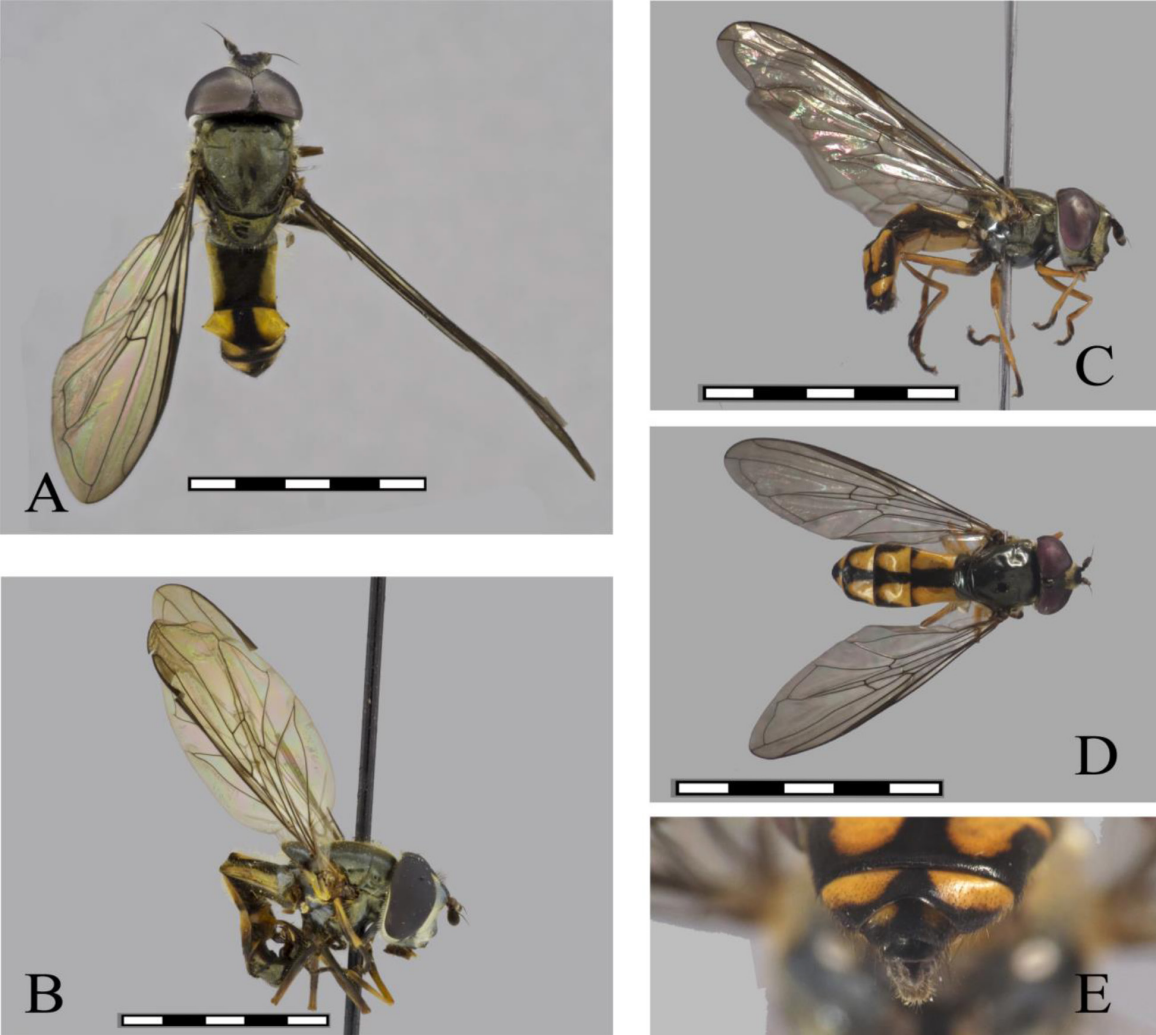
*Talahua fervida*, male (CEUA 95345): **A** dorsal view, male **B** lateral view (Paratype USNM). Female (CEUA 93328): **C** lateral view **D** dorsal view **E** posterior view, detail of maculae on sixth tergum. Scale bars: 5 mm.

**Figure 14.**
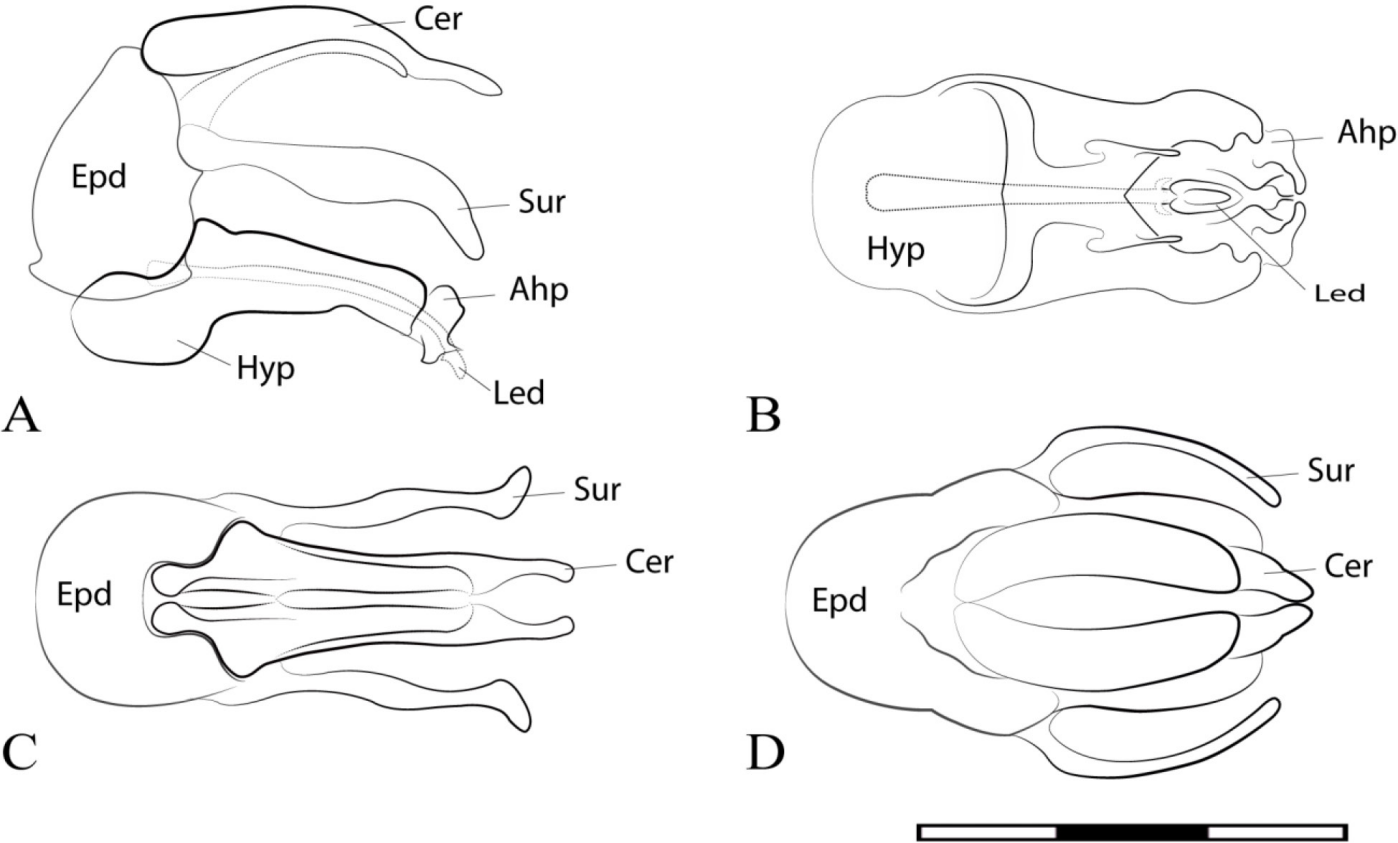
*Talahua fervida*, male genitalia: **A** whole genitalia including epandrium, cercus and surstylus, lateral view **B** hypandrium, dorsal view **C** cerci, and surstyli, ventral view **D** cerci and surstyli, dorsal view. Scale bar: 0.05 mm.

Figures 12 , 13 , 14 , 17

Melanostoma fervidum : Fluke (1945: 22).
Talahua fervidum : Fluke (1957: 262).
Talahua fervida : Thompson et al. 1976: 45 (catalog citation); Thompson 1999: 338 (catalog and taxonomic notes); Mengual et al. 2008: 545 (taxonomic list); Montoya 2016: 462 (catalog citation); Marín-Armijos et al. 2017: 184 (catalog citation).

##### Type specimen of

***Talahua fervida***

##### Fluke 1945.

**Holotype**. ♂, ECUADOR, Bolívar, Hda. Talahua. Original label: “ECUADOR, Bolivar, Hda. [Hacienda] Talahua, 3100 m a.s.l., 28.iv.1939, F.M. Brown & H. Brown collectors, AMNH”. / *Melanostoma* / (*Talahua*) / *fervidum* / Fluke” [red, handwritten except first line]”. The holotype is deposited at the AMNH in New York, USA. **Paratype** 3 ♂, same information as holotype, deposited in the AMNH, USNM, and WIRC (http://research.amnh.org/iz/types_db/details.php?specimen_id=2724, http://syrphidae.myspecies.info/taxonomy/term/140).

##### Genus differential diagnosis

(modified from Fluke 1945, Thompson and Skevington 2014). Male dichoptic. Both sexes with face slightly receding to perpendicular with a well-rounded tubercle, facial pollinosity yellowish (broadly punctuate in the *Platycheirus stegnus* species group or Carposcalis subgenus as well as some Argentinomyia species), never with transversal grooves dorsally along tubercle (present in some *Argentinomyia*); antennal pits distinctly separated (confluent in *Xanthandrus*); basoflagellomere large, slightly oval and apically rounded, scape broader than long, nearly equal to pedicel; metespisternum bare (with several fine subappressed hairs in *Xanthandrus*); katepisternal pile patches broadly separated throughout (broadly separated posteriorly, joined anteriorly in *Xanthandrus*); metasternum entire (greatly reduced in *Melanostoma*), bare; mesocoxa pilose posteriorly (bare in *Argentinomyia* and *Melanostoma*); metacoxa with a tuft of pile at posteromedial apical angle (bare in *Argentinomyia*); scutellum with a deep groove next to the rim (present in the new large *Argentinomyia* species described here); wing without maculae along the anterior edge of apical marginal cells; male legs, simple, slender, without bristles, pile tufts or modified hairs (modified, either broadened, or with special bristles, pile tufts or modified hairs as in *Platycheirus* and some *Tuberculanostoma*); tibiae usually yellow with a dark ring near the middle, more prominent on the metalegs; abdomen elongated or with parallel sides, with 4 to 5 pairs of large rounded to triangular maculae; male genitalia large, surstyli elongated, three to four times longer than broad and not forked, black; superior lobes elongate, no sickle-shaped; cerci elongate and yellow, “chitinous box” (= apex of hypandrium= superior lobes) elongated; lingula absent; aedeagus simple, no segmented, without apicomedial teeth.

##### Redescription

(modified from Fluke 1945: 22, and Mengual 2014). **Body size.** Large-sized flies, 9–12 mm. MALE. **Head** (Fig. 13A–C, E). Face large, shining black, wider than the thorax and abdomen, straight to perpendicular, not produced, with a large well-rounded tubercle (low dorsally, not distinct in some *Argentinomyia*), densely pollinose, sides of face usually with coppery reflections and a faint, slightly rugose area; front not swollen; gena large; oral tips, ocellar triangle, and the large triangular macula on the front shining black; pile on front generally black, on gena and face yellow to white, on ocellar triangle black, on the occiput yellow except the dorsal pile black; eyes bare, holoptic in male, with eyes contiguity as long as ocellar triangle; antennae black, pedicel and the lower basal corner of basoflagellomere red, short, scape broader than long, nearly equal to pedicel, basoflagelomere large, rounded or oval, ratio 1.1:1.5:2.5, arista black, dorsobasal, as long as the basoflagellomere or more, bare. **Thorax** (Fig. 13A–F): Black; postpronotum (humerus) bare; notopleuron with distinctive tubercle; scutum shining, covered with yellow pollen and short golden pile with many longer black pile that appear yellowish at the base, these pile become longer posteriorly; pleura yellow pollinose and pilose; scutellum black and with transversal rugose area on the disc, generally with longer yellowish and black setae on the posterior margin; subscutellar fringe complete, with multiple rows yellow pile; postmetacoxal bridge incomplete; pleura black to orange, whitish pollinose and pilose. *Legs* (Fig. 13C). Black; profemora yellow on the apical 1/3, mesofemora yellow on the apical 1/3, metafemora yellow on the apical 1/3; all the tibiae yellow with a dark ring near the middle, more prominent on the metatibia; tarsi brown, the pile yellow, black on the metatibia and above on the tarsi. *Wing* (Fig. 13A–F). Smoky; membrane entirely microtrichose, except for extensive bare areas on basal half (cells c, sc, r_1_, dm, and bm); the stigma and costal cell brownish; vein R_4+5_ straight; vein M_1_ (apical crossvein) oblique, slightly sinuous; alula broad, broader than cell cup, extensively bare,; calypter yellow, plumule simple, yellow; halter yellow, with a darkened capitulum. **Abdomen** (Fig. 13B–F). Elongated, black, with parallel sides, as broad as or broader than thorax, segments more or less quadrate, as long as broad, without premarginal sulcus, markings on tergites variable, with four pairs of lateral rounded to triangular prominent yellow maculae or with a complete basomedial black maculae; the first tergum shining, second with broad lateral yellow and only the apex black, third tergum with similar but wider maculae, fourth tergum with still wider but less elongate maculae, fifth tergum with a pair of small maculae in the basal corners. Pile yellow on the sides basally, black down the middle and on the apical terga. *Male genitalia* as Figs 12E, 14.

**Female.** (Fig. 13C–E). Similar to male except for normal sexual dimorphism. Abdominal maculae are comparatively shorter than in the male. Front narrow above, not the much wider than the ocellar triangle, shining above with a pollinose transversal macula below. Sixth tergum with a pair of small basolateral maculae. Sterna extensively yellow, yellow pilose, only brownish pilose in the apical corners of sterna four to sixth.

##### Distribution.

*Talahua fervida* is exclusively restricted to the Tropical Andes of Central and Occidental Cordillera in Colombia (Antioquia, Boyacá, Cundinamarca, Tolima) to Central Cordillera in Ecuador (Bolívar, Sucumbios). The species has a mountainous distribution in the biogeographical provinces of Cauca, Magdalena and North Andean Páramo (Fig. 17).

##### Additional material examined.

COLOMBIA: Antioquia, Bello, San Félix, Las Baldías, 6°20.029'N, 75°39.263'E, 2950–3150 m a.s.l., Net, 22.ii.2015; A. L. Montoya Leg. (1 ♀, CEUA 92108); Belmira, Páramo Santa Inés, Cabaña Cabildo Verde, El Morro-Alto de La Gallina, 6°40.167'N, 75°40.136'E, 3247 m a.s.l., Net in Clusia cf. brachycarpa Cuatrec., 4–14.ii.2017, A. L. Montoya; J. Sanchez; E. Orozco-G Leg. (1 ♂, CEUA 95345); Medellín, Corregimiento San Sebastián de Palmitas, Vereda La Volcana, High part, 6°21.232'N, 75°40.883'E, 2569–2650 m a.s.l., Van Sommeren-Rydon trap baited with fish, 22.ix.2011, L. Ríos-M Leg (1 ♀, CEUA 93328); San José de la Montaña, Vereda El Congo, Sector La Laguna, 6°46.013'N, 75°41.979'E, 3100–3183 m a.s.l., Net, 21–30.vi.2017, C. Henao; A. F. Sepúlveda Leg (1 ♀, CEUA 98074); Sonsón, San Francisco, Las Palomas A Mountain hill, 5°43.924'N, 75°15.444'E, 2749 m a.s.l., Forest, Net, 1-12.ix.2018, A.M. Echeverry, J. Vallejo Leg. (1 ♀, CEUA 103636); Boyacá, Flora and Fauna Sanctuary Iguaque, Ravien Carrizal, Cabaña Mamarramos, Lagunillas, 5°41.783'N, 73°26.516'E, 2850–3380 m a.s.l. (IAvH, in Gutiérrez et al. 2006). ECUADOR: Sucumbios, Santa Barbara, 0°37.868'N, 77°31.207'E, 3023 m a.s.l., 14.iii.1994, Gonorre Leg (1 ♂, QCAZ 103712).

##### Ecology.

Adults of *Talahua fervida* are found in highland ecosystems including cloud forests of the Andesand Páramo from 1800 to 3350 m a.s.l. The species has been associated with flowers of Clusia cf. brachycarpa Cuatrec (Clusiaceae Lindl.), but the immature stages are unknown.

**Figure 15.**
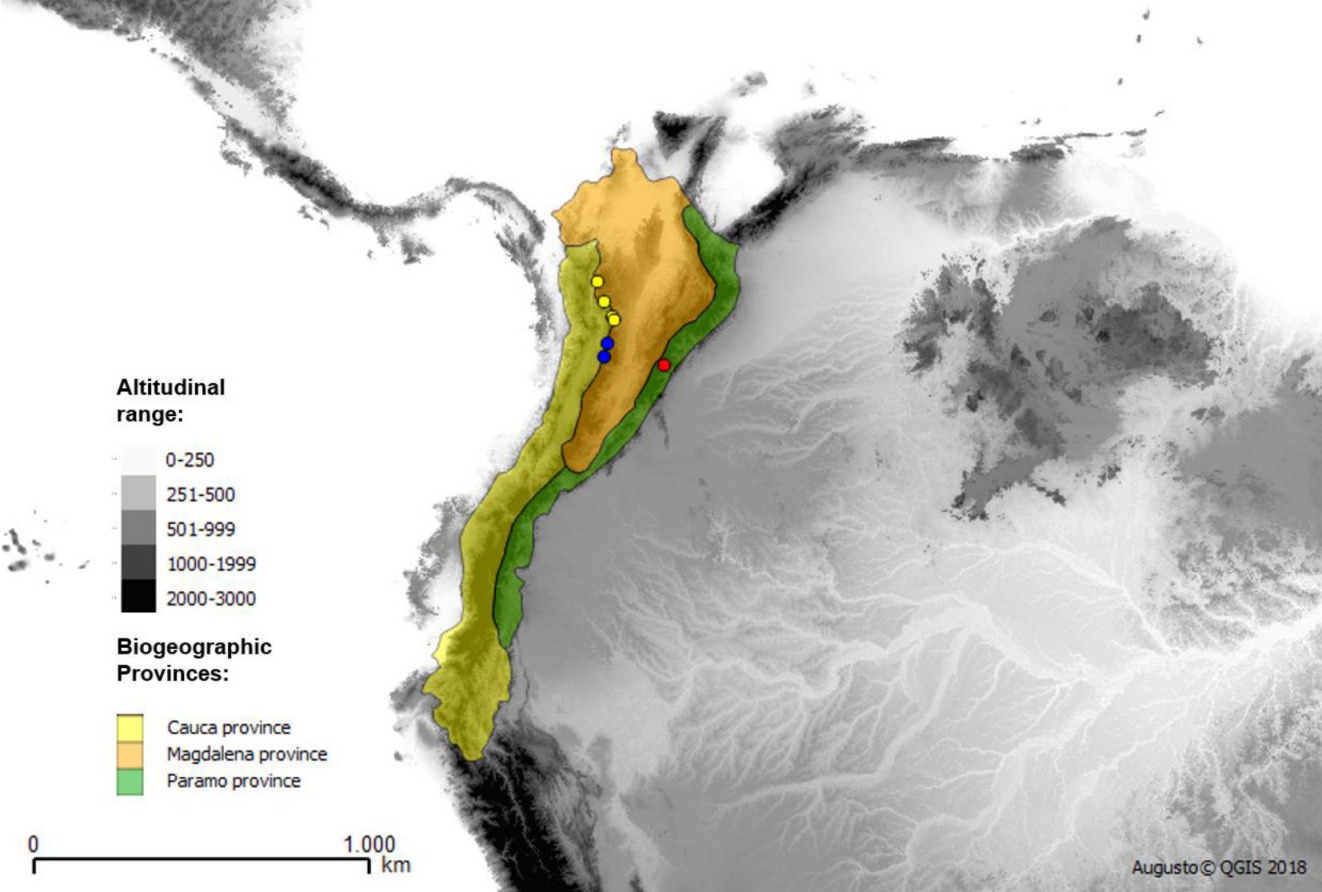
Biogeographical distribution of *Argentinomyia andina* sp. nov. (yellow), *A. quimbaya* sp. nov. (blue) and *A. choachi* sp. nov. (red).

**Figure 16.**
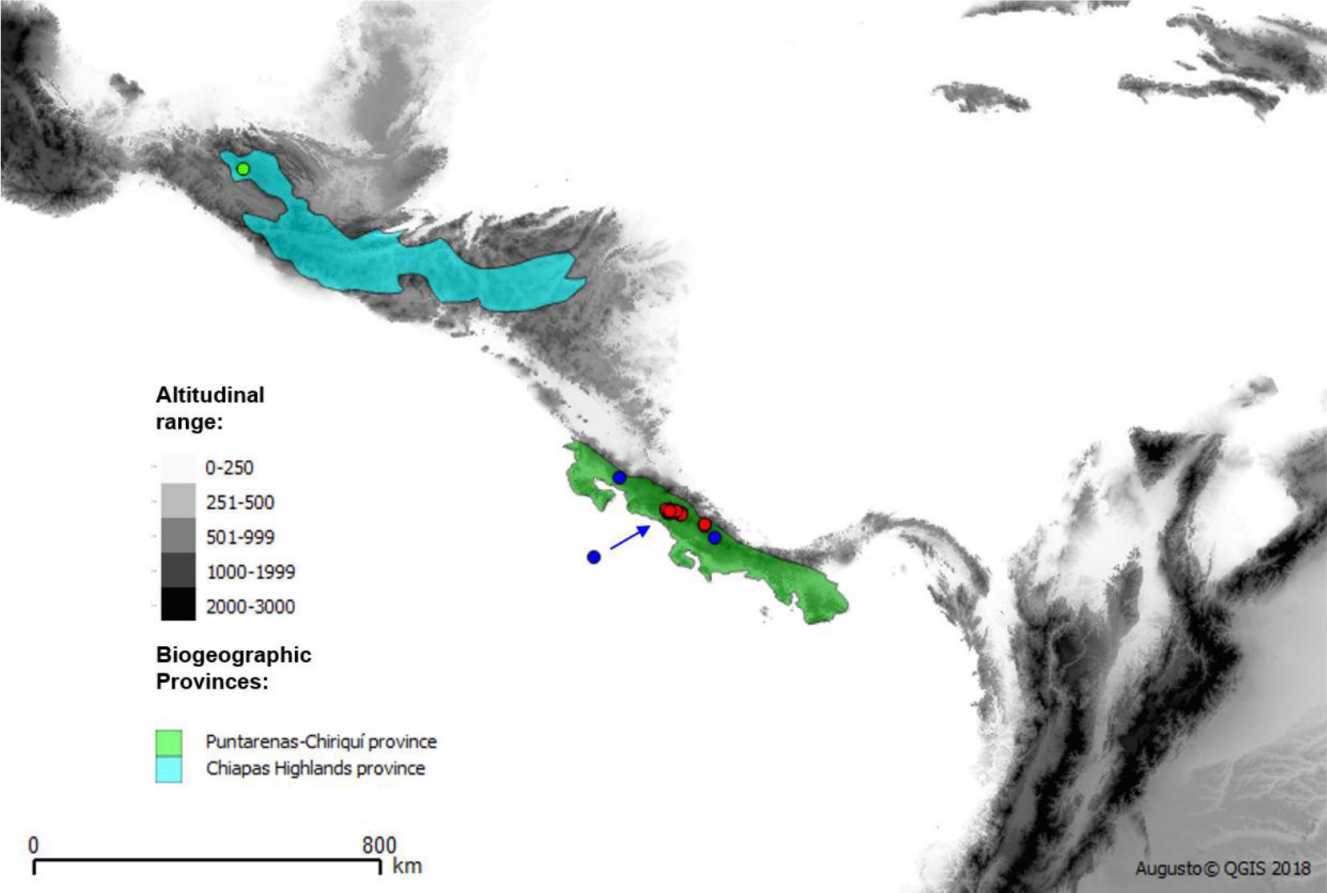
Biogeographical distribution of *Argentinomyia huitepecensis* sp. nov. (green), *A. talamanca* sp. nov. (red) and *A. puntarenas* sp. nov. (blue).

**Figure 17.**
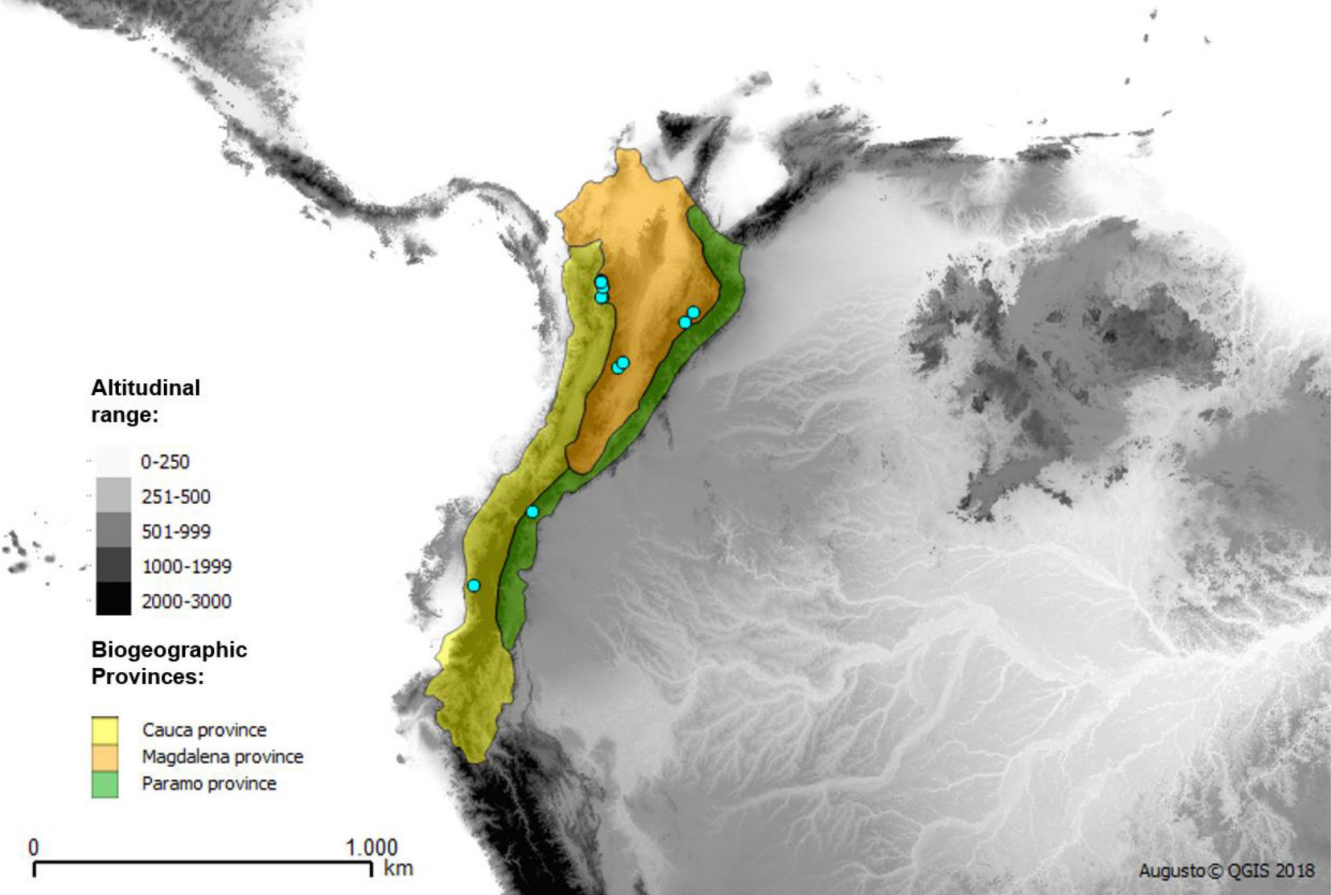
Biogeographical distribution of *Talahua fervida* (light blue).

## Discussion

The new *Argentinomyia* species described here can be distinguished from its congeners by the combination of the following characters: the basoflagellomere large, slightly oval and apically rounded; face with a well-rounded tubercle, never with transversal grooves dorsally along tubercle or broadly punctuate laterally; scutellum with a deep groove next to the rim (emarginate); metacoxa with pile posteromedial on apical angle; abdomen elongated or parallel sides with large markings. *Talahua fervida* differs from the new species by having the male genitalia large, including the surstyle, superior lobes, and cerci elongated, character recognized by Fluke (1957) and Thompson (1999) as exclusive for *Talahua*.

The new *Argentinomyia* species described here as well as *Talahua fervida* inhabit the Andean cloud forests and Páramo in Mesoamerica (México and Costa Rica) and Tropical Andes (Colombia and Ecuador) including five biogeographical provinces, which have been commonly referred to as hotspots of biodiversity (Maps 1, 2 and 3) (Myers et al. 2000). The distribution patterns suggest the existence of new endemic species in the highlands of neighboring countries.

Their restricted distribution, the local abundance and the fact that most species inhabit Protected and Conserved Areas suggest their vulnerability as proposed for several syrphid groups (see Montoya et al. 2012, Morales et al. 2013). This, besides the fact that many of these areas are facing anthropogenic pressures such as deforestation by mining, and wood extraction, indiscriminate and constant application of herbicides and pesticides, as well as the loss of biological corridors derived from the construction of hydroelectric and roads, highlight the risk for the survival of these species, as well as the maintenance of ecosystem services they provide.

In consequence and given that only one Neotropical species has been assessed in the IUCN Red List (Alaniz et al. 2018, López-García et al. 2019), there are compelling reasons to propose the new species as well as *Talahua fervida* as flagship entities for the conservation of the areas where they occur, been critical as environmental quality bioindicators. We considered that the information provided will constitute a baseline to assess their conservation status following the guidelines of the International Union for Conservation of Nature (IUCN).

## Supplementary Material

XML Treatment for Argentinomyia andina

XML Treatment for Argentinomyia choachi

XML Treatment for Argentinomyia huitepecensis

XML Treatment for Argentinomyia puntarena

XML Treatment for Argentinomyia quimbaya

XML Treatment for Argentinomyia talamanca

XML Treatment for Talahua

XML Treatment for Talahua fervida

## Supplementary materials

Supplementary material 1 Table S1 Data type occurrence https://binary.pensoft.net/file/402772This dataset is made available under the Open Database License (http://opendatacommons.org/licenses/odbl/1.0/). The Open Database License (ODbL) is a license agreement intended to allow users to freely share, modify, and use this Dataset while maintaining this same freedom for others, provided that the original source and author(s) are credited.Augusto L. Montoya, Marta Wolff
